# *Acer truncatum* Bunge seed oil attenuates learning and memory impairment in AD mouse model via modulating gut microbiota and metabolism

**DOI:** 10.3389/fnut.2026.1757330

**Published:** 2026-02-10

**Authors:** Duoduo Ren, Xuejun Chai, Chenyao Xiang, Yongkang Zhao, Penghao Sun, Mengli Wang, Jing Li, Jiayue Wu, Chenju Yi, Shulin Chen, Enyao Li, Shanting Zhao

**Affiliations:** 1 College of Veterinary Medicine, Northwest A&F University, Yangling, China; 2 College of Basic Medicine, Xi'an Medical University, Xi'an, China; 3 Kunming Institute of Zoology, Chinese Academy of Sciences, Kunming, China; 4 Research Center, Seventh Affiliated Hospital of Sun Yat-sen University, Shenzhen, China; 5 Department of Children Rehabilitation, The Fifth Affiliated Hospital of Zhengzhou University, Zhengzhou, China

**Keywords:** *Acer truncatum* Bunge seed oil, Alzheimer's disease, fecal microbiota transplantation, gut microbiota, serum metabolomics, short-chain fatty acids

## Abstract

This study aimed to clarify the neuroprotective effect of *Acer truncatum* Bunge seed oil (ASO) and its interactions with the gut microbiota in transgenic mice with 5 × Familial Alzheimer's disease (5 × FAD). The AD-transgenic mice were fed with standard diet supplemented with 4% ASO from one to six months of age. The result show that ASO intervention can alleviate learning and memory impairment, enhance motor coordination and endurance, and reduce Aβ deposition in the brains. It also inhibit the proliferation of microglia and astrocytes, decrease the levels of IL-1β, IL-6, and TNF-α in the hippocampus and serum. Then, ASO could increase the Chao1 index and Shannon index, alter the gut microbiota composition, specifically, enhance the growth of gut bacteria correlated with the production of SCFAs, including *Ruminococcaceae, Butyricicoccus, Sutterella* and others, particularly those related to butyrate production. Additionally, ASO can increase the concentrations of SCFAs in fresh feces and serum, particularly butyric acid. ASO could primarily modulate the biosynthesis of unsaturated fatty acids, glycerophospholipid metabolism, and sphingolipids metabolism in serum. At the same time, Fecal microbiota transplantation (FMT) could reduce Aβ deposition, enhance learning and memory. Finally, Supplementation of sodium Buty also mitigate learning and memory impairments. This study highlights the gut microbiota might be a potential therapeutic target for AD and provides a scientific foundation for developing novel pharmaceuticals or nutraceuticals.

## Introduction

Alzheimer's disease (AD) is a progressive neurodegenerative disorder with multifactorial causes, currently lacking effective treatment options. China is among the countries with the largest and fastest-growing elderly population globally, also identified as a high-risk nation for AD. The incidence of AD continues to rise in parallel with the aging population. Consequently, early prevention of AD emerges as one of the most critical endeavors in healthcare today. As the most common form of dementia, AD predominantly affects older adults, comprising 60–80% of cases (1). Its hallmark is gradual deterioration of cognitive abilities and memory (2). Accumulating evidence supports that AD is primarily characterized by β-amyloid (Aβ) plaques deposition and the formation of neurofibrillary tangles largely composed of hyperphosphorylated tau protein (3, 4). Additionally, crucial features such as neuroinflammation, synaptic dysfunction, neuronal loss, autophagy dysfunction, gut microbiota dysbiosis, and metabolic dysregulation play a vital roles in the development and progression of physiological impairments in AD (5–10). Over past decades, numerous monotherapies focusing on Aβ, tau, or neuroinflammation have failed to stop or slow AD progression, highlighting the continued absence of effective disease-modifying therapies (11). Therefore, finding new therapeutic methods and developing safe and effective drugs remain central to current research.

Research points to gut microbiota and their derived metabolites as key contributors to AD (12). Growing evidence unveils the gut microbiome and the central nervous system (CNS) interact bidirectionally via the “microbiota-gut-brain axis (MGBA)” (13, 14). Multiple pathways mediate communication between the gut microbiota and the CNS despite anatomical separation. This involves regulation of the circulatory system, immune system, enteric nervous system, vagus nerve, and neuroendocrine system through neuroactive compounds, metabolites and hormones (15). The MGBA has emerged as a novel approach for AD therapy, although the mechanisms by which gut bacteria influence the brain remain unclear (16). Extensive research indicates that aging, a predominant risk factor for AD, is associated with progressive intestinal barrier breakdown. This process enables gut microbiota-derived inflammatory metabolites to enter the bloodstream circulation, inducing systemic inflammation, blood-brain barrier (BBB) disruption, and subsequent CNS degeneration (17, 18). Recent evidence show that neuropathological alterations in AD brains negatively impact the gut microbiota and intestinal barrier function (19, 20). Disrupted gut barrier function allows bacteria and their metabolites to enter circulation, aberrantly stimulating immune responses and contributing to systemic inflammation, CNS inflammation, and degeneration (21). Consequently, a potential AD therapy involves regulating gut homeostasis by suppressing proinflammatory and enhancing anti-inflammatory microbial metabolism (22). The MGBA offers actionable perspectives for achieving this objective to prevent and delay AD (23). Fecal microbiota transplantation (FMT) has shown beneficial effects in AD therapy. Recent research demonstrates that FMT therapy and short-chain fatty acids (SCFAs) intervention, particularly sodium butyrate (Buty), significantly decrease Aβ accumulation, ameliorate tau pathology, inhibit neuronal damage and apoptosis, and improve learning and memory in AD mice, playing a crucial role in maintaining microbiota-gut-brain health (24–26). Therefore, discovering new therapeutic methods and safe, effective drugs is urgently needed.

Natural products have a long-standing history in the prevention and therapy of diseases (27, 28). *Acer truncatum* Bunge, commonly known as yuanbaofeng, or yuanbaoqi, is a tree species of the genus *Acer*, within the *Sapindaceae* family. Traditionally, it has functioned as a medicinal, edible, and ornamental plant in northern China (29, 30). *Acer truncatum* Bunge seed oil (ASO) was approved as a novel food resource by the People's Republic of China's Ministry of Health in 2011 (31). ASO comprises long-chain monounsaturated fatty acids (MUFAs), and long-chain polyunsaturated fatty acids (PUFAs), and saturated fatty acids (SFAs) (32). Long-chain unsaturated fatty acids are categorized as Omega-3, Omega-6, and Omega-9 fatty acids (FAs) according to double bond position, all of which are present in ASO (33). FAs, essential for human growth and physiological function, are closely linked to brain health (34). Omega-3 PUFAs, including α-linolenic acid (ALA, C18:3, ω-3), eicosapentaenoic acid (EPA, C20:5, ω-3), and docosahexaenoic acid (DHA, C22:6, ω-3), increase synaptic plasticity, decrease neuronal loss, and demonstrate anti-inflammatory activity linked to improved cognition (35). Omega-6 PUFAs, such as linoleic acid (LA, C18:2, ω-6) and γ-linolenic acid (C18:3, ω-6), participate in neuronal membranes formation and offer therapeutic potential for chronic, and autoimmune neurodegenerative diseases (36). Omega-9 MUFAs include oleic acid (OA, C18:1, ω-9), erucic acid (EA, C22:1, ω-9), and nervonic acid (NA, C24:1, ω-9), with NA as the key FA in the nervous system (37). NA was first discovered in the shark brain in the form of nervonic sphingolipids (SLs), where it is attached to the sphingosine backbone via an amide bond (38). Serving as a significant component of SLs in myelin membranes, NA is critical for the growth and maintenance of neural tissues (39). As the principal long-chain FA in lipids such as phosphatidylcholine (PC), sphingomyelin (SM), and ceramide (Cer), NA's synthesis serves as the rate-determining step in maintaining myelin sheath lipid homeostasis (40). Research indicates that NA supplementation regulates the expression of phosphatidylinositide 3-kinases (PI3K), protein kinase B (Akt), mammalian target of rapamycin (mTOR), and downregulated the expression of interleukin-1β (IL-1β), interleukin-6 (IL-6), and tumor necrosis factor-α (TNF-α), and improves cognitive impairment in D-galactose/AlCl3 in mice (41). ASO is the principal plant resource for mass production of NA, containing 3–7% NA (33). ASO intake was found to primarily influence sphingolipid, glycerolipid, and glycerophospholipid metabolism pathways in serum and brain (42). Recently our research group found that ASO supplementation mitigates cuprizone-induced myelin loss, highlighting its potential as a novel dietary therapy for demyelinating diseases (43). Therefore, ASO, rich in NA, serves as a functional food with neuroprotective potential.

In this study, we employed multidisciplinary approaches, including behavioral tests, fluorescent immunohistochmistry, gut microbiota profiling, SCFAs analysis, metabolomic analysis, transcriptomic analysis, and additional experimental assays to investigate the efficacy of ASO on transgenic mice with 5 × FAD. These effects encompass learning and memory enhancement and reduction of Aβ accumulation. Notably, our findings suggest that these therapeutic benefits may be achieved through multiple mechanisms: modulation of gut microbiota, regulation of unsaturated FAs biosynthesis, glycerophospholipid, sphingolipid metabolism, inhibition of neuroinflammatory responses, and alleviation oxidative stress. Consequently, the utilization of ASO as a component in pharmaceutical formulations and dietary supplements presents a potential strategy for AD prevention.

## Materials and methods

### Materials

The standard laboratory chow was acquired from Xietong Pharmaceutical Biotechnology Co., Ltd. (Jiangsu, China). Mouse IL-1β (F2040-A), mouse IL-6 (F2163-A), mouse TNF-α (F2132-A) ELISA kits were obtained from Shanghai Kexing Trading Co., Ltd. (Shanghai, China). Human Aβ1–42 (E-EL-H0543), human Aβ1–40 (E-EL-H0542) were obtained from Elabscience Biotechnology Co., Ltd. (Wuhan, China). The glutathione peroxidase (GSH-Px; S0056), superoxide dismutase (SOD; S0101S) and reactive oxygen species (ROS; S0033S) kits were acquired from Shanghai Beyotime Biotechnology Co., Ltd. (Shanghai, China). All antibiotics and sodium Buty (CAS: 156-54-7) were purchased from Shanghai Aladdin Biochemical Technology Co., Ltd. (Shanghai, China). Other reagents were obtained from Sinopharm Chemical Reagent Beijing Co., Ltd.

### Analysis of ASO composition

ASO was refined by Shandong Fengzhishen Biological Co., Ltd. (Shandong, China) and stored at −20 °C until further use. The fatty acid composition were detected based on the Agilent 8890-7000E gas chromatography-tandem mass spectrometry (GC-MS/MS) platform. GC Conditions: the sample derivants were analyzed using an gas chromatography (Agilent 7890B) couple to triple quadrupole mass spectrometry (Agilent 7000D). The GC analytical conditions were as follows, the separation was conducted on a DB-5MS capillary column (30 m × 0.25 mm × 0.25 μm, Agilent) with high purity helium (purity >99.999%) as carrier gas. The heating procedure was started at 40 °C and held for 2 min, increased to 200 °C at a rate of 30 °C/min and held for 1 min, increased to 240 °C at a rate of 10 °C/min and held for 1 min, increased to 285 °C at a rate of 5 °C/min and held for 3 min. The flow rate was set at 1 ml/min. The injection volume was set at 1 μl with splitless. The inlet temperature was set at 250 °C. MS Conditions: the sample derivants were analyzed using an gas chromatography (Agilent 7890B,) couple to triple quadrupole mass spectrometry (Agilent 7000D,). The mass spectrometry parameters can be set below. The temperature and ionization voltage of electron ionization source (EI source) was 230 °C and 70eV, respectively. The transmission line and quadrupole temperature was 280 °C and 150 °C. The sample data was acquired in SIM mode after solvent delay for 4 min.

### Animal ethical statement

C57BL/6J mice (6–8 weeks old) were obtained from SPF Biotechnology Co., Ltd., Beijing, China (production license No. SCXK 2019-0010; qualification No. 110324230102145121). Male 5 × FAD-transgenic mice (Stock No: 006554) on a congenic C57BL6 background were sourced from the Jackson Laboratory (Bar Harbor, USA). These 5 × FAD mice were provided by Professor Chenju Yi of Sun Yat-sen University (Shenzhen, China). The 5 × FAD mice were periodically backcrossed with female C57BL/6J mice, and the offspring were genotyped, via PCR conducted on DNA derived from ear tissue (44, 45). Thy1-YFP transgenic mouse line was provided by Professor Shengxiang Zhang of Lanzhou University (Lanzhou, China). All mice were housed in standard conditions (22–24 °C, 50–60% humidity) under a light and dark cycle for 12/12 h, with free access to standard diet and water. Every effort was made to ensure optimal animal welfare, minimize discomfort, and limit animal use. The Care and Use of Laboratory Animals (ISBN-10: 0-309-15396-4) was used to carry out all experiments. The study was approved by the Animal Ethical and Welfare Committee, Northwest A&F University (Yangling, China; Approval No. 2023065) and performed following the Care and Use of Laboratory Animals by Northwest A&F University.

### Animal experimental design

Experiment 1 (ASO intervention): to assess the impact of ASO on 5 × FAD mice, 20 male 5 × FAD mice were randomly divided into two groups: (A) AD group (*n* = 10) and (B) ASO supplementation (AD-ASO) group (*n* = 10). Additionally, 10 age-matched male littermates wild-type (WT group) served as controls. The WT and AD groups received standard diet, while the AD-ASO group was fed with standard diet supplemented with 4% ASO from one to six months of age. All other rearing conditions remained consistent across groups. At the experiment's conclusion, eye frame blood was collected in mice, then centrifuged at 3,000 rpm for 15 min at 4 °C, and stored at −80 °C for metabolomics and other analysis. Subsequently, mice were dissected, and colon contents samples were aseptically obtained and preserved at −80 °C for gut microbiota analysis. Hippocampus, cortex, and other biological samples were obtained and maintained at −80 °C for further analysis. Additionally, three mice/group were perfused with 0.9% saline followed by 4% paraformaldehyde (PFA) in 0.1 M PB for subsequent immunofluorescent histochemistry.

Experiment 2 (FMT and Sodium Buty intervention): three-month-old male 5 × FAD mice and their litter control WT mice were randomly assigned to three groups: (A) AD group (*n* = 10); (B) AD-FMT group (*n* = 10); (C) WT group (*n* = 10); and (D) AD-Buty group, (*n* = 10). FMT was performed to determine whether the gut microbiota play a causal role in mediating ASO's effects on learning and memory deficits and Aβ deposition in 5 × FAD mice. To achieve substantial depletion of the gut microbiota prior to FMT, standard-diet mice were exposed to an antibiotic cocktail (Abx) administered via drinking water for three consecutive days. The cocktail consisted of metronidazole (100 mg/L), penicillin (100 mg/L), neomycin (100 mg/L), vancomycin (50 mg/L), and streptomycin (50 mg/L) (46, 47). For preparing FMT material, fresh fecal samples were obtained from 3-month-old 5 × FAD mice fed with 4% AD-ASO as donor mice after 28 days. Fecal samples were promptly pooled, suspended in sterile PBS (1 g feces/10 ml PBS), and homogenized. Following centrifugation at 500 rpm for 5 min at 4 °C, the supernatant was obtained. The supernatant (150 μl) was orally administered to antibiotic-pretreated mice once daily for 28 days (AD-FMT group). In the Buty intervention, AD-Buty group mice received sodium Buty (Aladdin, China) in drinking water at a concentration of 0.1 M for 28 days.

### Behavioral test

Morris water maze test (MWM): the MWM test was employed to evaluate the spatial learning and memory of the mice. In summary, a round water maze was partitioned into four equal quadrants, with a hidden platform positioned at the center of the target quadrant. The initial five days were dedicated to positioning navigation tests, during which the escape latency of mice locating and ascending the hidden platform was recorded. On the sixth day, the platform was removed, and a exploration test was conducted, tracking the mice's trajectory over a 60 s period.

Novel object recognition test (NOR): the NOR test was employed to evaluate memory and cognitive function, specifically the ability to recognize and differentiate between novel and familiar objects (48). The open field measured 55 × 55 × 40 cm, with its base divided into 25 squares (11 × 11 cm). The apparatus was verified to be clean and odorless. On the first day, a habituation phase (pre-test) was conducted to minimize stress and acclimatize the mice to the experimental environment. Mice were released into the empty open field for a duration of 5 min, allowing it to explore and familiarize itself with the surroundings. On the second day, the sample phase (training phase) allowed the mice to explore the objects and form a memory of the familiar ones. This phase comprised an exploration of two identical objects (designated as the old object, a red cube) placed in an arena within the open field for 5 min. The formal testing phase aimed to assess the mouse's ability to recognize a novel object. Each mouse was examined in an open field with two consecutive test phases separated by a 6 h interval. One of the original objects (a red cube) was switched to a new object (a green cylinder) for testing. Mice explored the objects for a 5-min session. The time spent exploring the new object and the old object in the formal testing phase was recorded. The recognition index = time spent exploring the new object/(total time spent exploring both objects).

Rotarod test: rotarod treadmill test was employed to assess locomotor coordination and endurance of mice. A longer duration on the rotating treadmill indicated superior coordination and endurance. Mice trained at 20 rpm/min for 3 min over two days and were tested at 40 rpm/min on the third day at the same time. Trials ended automatically upon falling or after 5 min.

Grip and string tests: the grip and string tests were employed to assess coordination, endurance, and physical performance of mice (49). The apparatus comprised a horizontal metal rod, 3 mm in diameter and 60 cm in length, positioned 40 cm above the table surface. Mice were suspended by their tails, allowing their forepaws to contact the middle of the rod. The duration of their stay on the rod was measured, with a maximum time limit of 30 s. For the grip test, a three-point scoring system was utilized: zero points if the mouse failed to grasp the rod, one point for staying 1–10 s, two points for 11–20 s, and three points for 21 s or longer. The grip test scores were then aggregated, with a maximum possible score of three points. The string test evaluated the mouse's ability to hang and move from the stem over a 30-s period. The scoring system was as follows: zero points if the mouse fell within 30 s; one point if it hung using only two forelimbs for 30 s; score two points if it hung using all four paws; three points if it hung using four paws and its tail; four points if it hung using four paws and its tail while moving along the stem; and five points if it hung using four paws and its tail, moved along the stem, and reached one of the vertical rods at the stem's tip within 30 s. The maximum possible score for the string test was five points.

### Thioflavine S staining

Mice were perfused with 0.9% saline followed by 4% PFA, and brains were coronally sectioned into 50 μm slices using a vibratome (VT1000S, Leica, Germany). Thioflavine S (TS) was utilized to visualize amyloid plaques (50). Images were acquired using a fluorescence microscope (Axio Obser Z1, Zeiss, Germany). Quantification of positive objectives was conducted by using Image J analysis software (version 1.52).

### Immunofluorescence

The brain was coronally sectioned at 50 μm using a vibratome. For immunohistochemistry, sections were incubated overnight at 4 °C with primary antibodies diluted in blocking solution. Primary antibodies were as follows: rabbit anti-GFAP (1:500, Santa Cruz Biotechnology, California, USA) and rabbit anti-Iba1 (1:1,000, Abcam). Then incubated in species-specific secondary antibodies (1:500 for all) or 3 h at room temperature. Images were acquired using a fluorescence microscope. Quantification of positive objectives was conducted by using Image J analysis software.

### Determination of inflammatory cytokines and oxidative stress

The hippocampus from each group of mice was weighed, and a corresponding volume of PBS was added relative to the weight. Samples were homogenized and centrifuged (3,500 rpm/min, 15 min) at 4 °C. Then, the supernatant was collected. The IL-1β, IL-6, and TNF-α concentrations were measured using ELISA kits following the manufacturer's protocol. Similarly, the measurement of GSH-Px, SOD, and ROS concentrations in hippocampus was performed. Additionally, the measurement of Aβ1–42 and Aβ1–40 concentrations in brain tissues were conducted using ELISA kits.

### 16S rRNA sequencing

High-throughput sequencing of total DNA from colon content samples was conducted by Shanghai Personal Biotechnology Co., Ltd. (Shanghai, China). Illumina sequencing was conducted as previously described (51). The V3-V4 region of the 16S rRNA gene was amplified by PCR using the forward primer (5′-ACTCCTACGGGAGGCAGCA3′) and the reverse primer (5′-GGACTACHVGGGTWTCTAAT3′). The ASV/OTU was analyzed using the quantitative analysis with the QIIME2 platform, R software (v.3.2.0), and the Greengenes database. Subsequent analyses were performed.

### Analysis of fecal and serum SCFAs

The samples of every mice were obtained, and SCFAs were assessed. Gas chromatography was performed on an Agilent HP-INNOWAX capillary column (30 m × 0.25 mm ID × 0.25 μm). Helium served as the carrier gas at 1 ml/min. Mass spectrum conditions: mass spectrometric detection of metabolites was performed on ISQ 7,000 with electron impact ionization mode. SIM mode was used with the electron energy of 70 eV.

### Serum metabolomics

The serum samples were sent to Shanghai Personal Biotechnology Co., Ltd. (Shanghai, China) for analysis. Raw data were converted to .mzXML format using ProteoWizard software. Subsequently, XCMS software was employed for peak alignment, retention time correction, and peak area extraction. The extracted XCMS data underwent a series of processes: metabolite structure identification, data preprocessing, experimental data quality evaluation, and finally, comprehensive metabolomics analysis.

### UID RNA-sequencing of hippocampal tissues

For RNA isolation, approximately 30 mg of hippocampal tissue was used. Seqhealth Technology Co., Ltd. (Wuhan, China) conducted the RNA extraction and sequencing process. Standard RNA-seq analysis was performed using de-duplicated consensus sequences. Gene set enrichment analysis (GSEA) was executed using pyGSEA, incorporating background datasets from GO (2018) and KEGG (2019).

### Real-time quantitative PCR

Hippocampus RNA extraction and RT-qPCR were performed following previously established protocols (52). Table 1 provides the sequences of the primers utilized.

**Table 1:** The RT-qPCR primers for the targeted genes in this study.

**Gene**	**Primer *F***	**Primer *R***
IL-1β	TGACGGACCCCAAAAGAT GA	TCTCCACAGCCACAATGAGT
IL-6	ACCGCTATGAAGTTCCTCTC	CTCTGTGAAGTCTCCTCTCC
TNF-α	AGTCCGGGCAGGTCTACT TT	GTCACTGTCCCAGCATCTTGT
GAPDH	AGGTTGTCTCCTGCGACTG CA	GTGGTCCAGGGTTTCTTACT CC

### Statistical analysis

Statistical analysis was conducted using SPSS (v.25.0) and GraphPad Prism (v.9.4.1), respectively. Behavioral tests, the analysis was conducted using SMART 3.0 software. One-way analysis of variance (ANOVA) followed by Tukey's test was adopted to compare different groups. Signficance levels are indicated as ^*^P < 0.05, ^**^P < 0.01, ^***^P < 0.001, and data were expressed as mean ± standard error of mean (SEM). Non-significant differences of note are denoted by “ns” in the figures.

## Results

### Component analysis of ASO

The component analysis of ASO was analyzed using GC-MS/MS. Primary components of ASO are detailed in Table 2, predominantly comprising FAs. These include LA (C18:2, ω-6) at 31.57%, OA (C18:1, ω-9) at 21.17%, EA (C22:1, ω-9) at 12.12%, palmitic acid (C16:0) at 10.72%, cis-11-Eicosenoic acid (C20:1, ω-9) at 8.51%, stearic acid (C18:0) at 4.85%, NA (C24:1, ω-9) at 3.02%, and additional FAs (Table 1). Analysis of ASO revealed FAs components of 44.82% for omega-9, 33.08% for omega-6, and 3.36% for omega-3.

**Table 2:** The component analysis of ASO by GC-MS/MS.

**Ingredients**	**Description**	**Content (%)**
Palmitic acid	C16:0	10.72
hexadecanedioic acid	C16:2	0.15
Heptadecanoic acid	C17-0	0.13
Stearic acid	C18:0	4.85
Oleic acid (OA)	C18:1ω-9	21.17
Linoleic acid (LA)	C18:2ω-6	31.57
α-linolenic acid (ALA)	C18:3ω-3	2.34
γ-linolenic acid	C18:3ω-6	0.74
Arachidic acid	C20:0	0.35
cis-11, 14, 17-eicosatrienoic acid	C20:3ω-3	0.13
cis-11-Eicosenoic acid	C20:1ω-9	8.51
cis-11, 14-eicosadienoic acid	C20:2ω-6	0.42
cis-5, 8, 11, 14, 17-eicosapentaenoic acid (EPA)	C20:5ω-3	0.56
Heneicosanoic acid	C21-0	0.11
Behenic acid	C22:0	0.73
Erucic acid (EA)	C22:1ω-9	12.12
cis-4, 7, 10, 13, 16, 19-docosahexaenoic acid (DHA)	C22:6ω-3	0.33
Tricosanoic acid	C23:0	0.37
Lignoceric acid	C24:0	0.47
Nervonic acid (NA)	C24:1ω-9	3.02
ω-9 FAs	—	44.82
ω-6 FAs	—	32.73
ω-3 FAs	—	3.36
Monounsaturated fatty acids (MUFAs)	—	44.82
Polyunsaturated fatty acids (PUFAs)	—	36.09

### ASO alleviated the impairment of learning and memory in 5 × FAD mice

**Figure 1:**
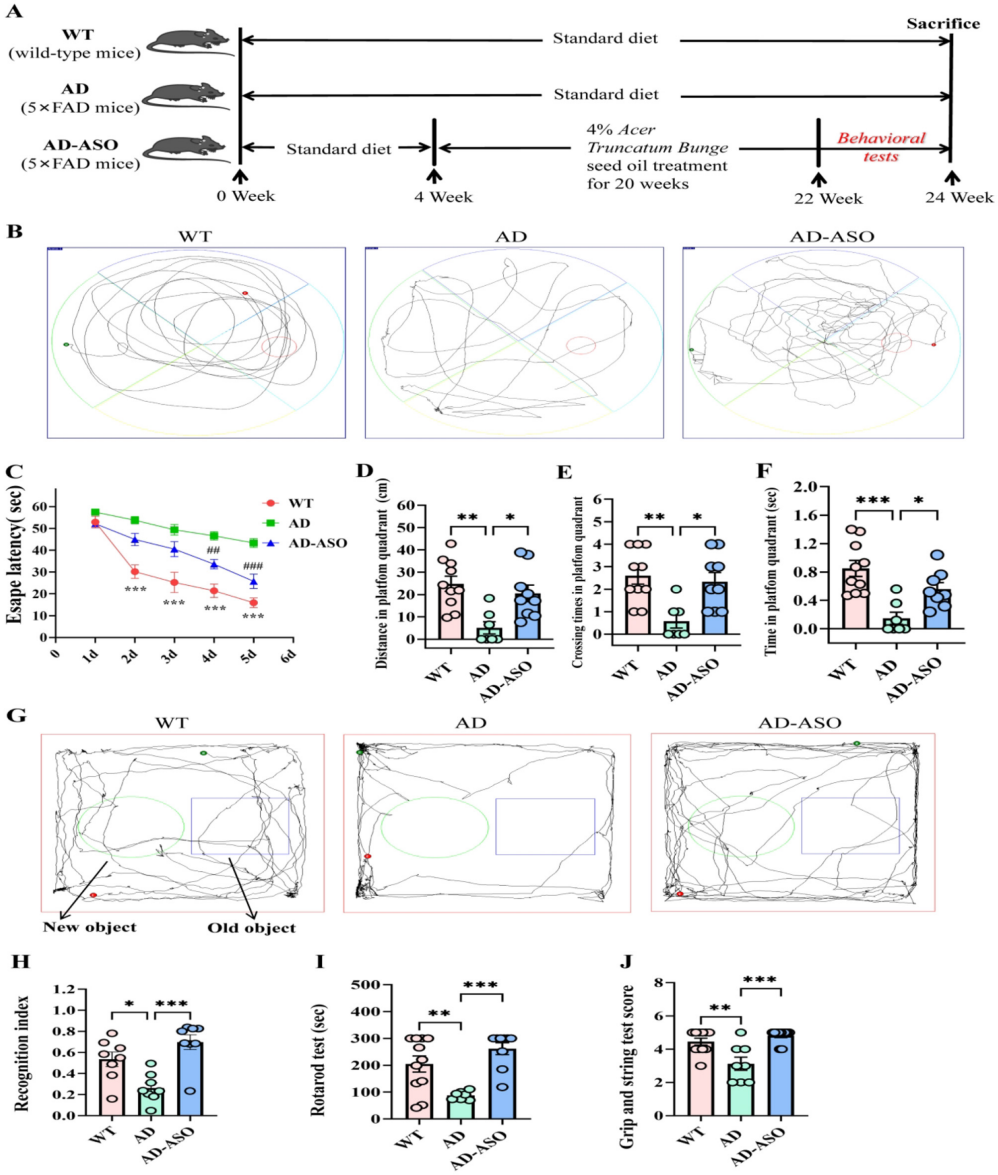
ASO alleviated learning and memory deficits of 5 × FAD mice. **(A)** The experiment workflow of animal-administration. **(B)** Representative track images of mice in the MWM test. **(C)** Escape latency during training days in MWM test (*n* = 10), ***P < 0.001, WT group compared to the AD group; ^##^*P* < 0.01, ^###^*P* < 0.001, AD-ASO group compared to the AD group; Data are presented as mean ± SEM, and statistical significance was determined by Two-way ANOVA with Tukey's test. **(D)** Distance in platfom quadrant. **(E)** Crossing times in platfom quadrant. **(F)** Time in platfom quadrant. **(G)** Representative track images of mice in the NOR test. **(H)** Recognition index in the NOR test. **(I)** Rota-rod test. **(J)** Grip and string test score. **(D–F)** and **(H–J)** data are presented as mean ± SEM (*n* = 10). *P < 0.05, **P < 0.01, ***P < 0.001. WT, wild-type control group; AD, 5 × FAD transgenic Alzheimer's disease model group; AD-ASO, 5 × FAD mouse *Acer truncatum* Bunge seed oil intervention group.

Figure 1A shows the animal experiment workflow. The MWM test to assess the potential of ASO in improving learning and memory in 5 × FAD mice. In the navigation test (Figure 1C), the AD mice exhibited longer escape latency in a circular motion to find the platform in the outer area on days 2–5 (*P* < 0.001), compared with the WT mice. Interestingly, compared to the AD group, mice administrated with ASO demonstrated shorter escape latency to find the platform on days 4–5 (Figure 1C, *P* < 0.01). Furthermore, in the probe trial conducted on day 6 (Figure 1B), ASO-administrated mice showed significantly higher number of cross-platforms, longer distance and time in the target quadrant compared to the AD mice (Figures 1D–F, *P* < 0.05). The data indicate that ASO-administration significantly improved the learning and memory of 5 × FAD mice. The NOR test, a commonly used behavioral test in mice, was applied to assess recognition. Figure 1G presents the representative track images of mice in the NOR test. Compared to the AD group, ASO-administrated mice exhibited improved recognition index (Figure 1H, *P* < 0.001). The results indicatet that ASO-administration significantly enhanced learning, memory and recognition of 5 × FAD mice.

Rotarod test is widely used to assess motor function, balance, and learning ability in rodents. Our findings revealed that ASO-administration significantly enhanced the motor coordination and endurance of AD mice (Figure 1I, *P* < 0.001). The grip and string test, a straightforward behavioral assessment, is employed to measure coordination, endurance, and physical performance of mice, proving particularly valuable in studies of neurodegenerative diseases. Our results indicate that ASO-administration significantly improved the coordination, endurance, and physical performance of AD mice (Figure 1J, *P* < 0.001). Collectively, the results indicate that ASO-administration effectively alleviated learning and memory impairment and enhanced motor coordination and endurance of 5 × FAD mice.

### ASO administration reduced Aβ deposition in the brains of 5 × FAD mice

**Figure 2:**
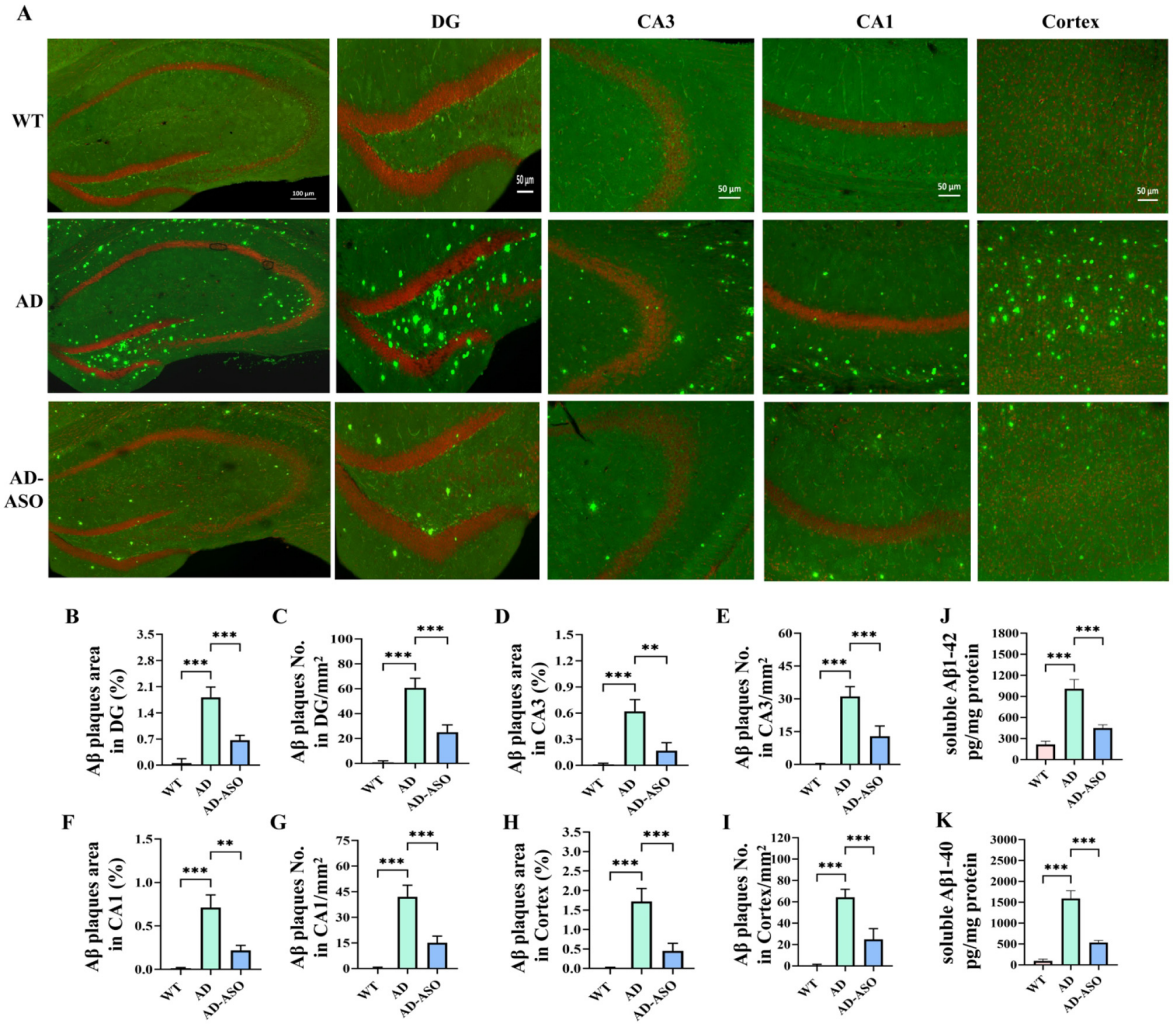
ASO administration alleviated Aβ deposition in 5 × FAD mice. **(A)** Representative images of TS staining in indicated brain regions (*n* = 9 brain sections, from three mice per group). **(B–I)** Quantification of the Aβ plaques (*n* = 9 brain sections, from three mice per group) in the DG, CA3, and CA1 subregions of hippocampus, and the cortex from indicated group. **(J, K)** Soluble Aβ1–42 and Aβ1–40 content in mouse brain (*n* = 6). Data are presented as mean ± SEM. **P< 0.01, ***P < 0.001. WT, wilde-type control group; AD, 5 × FAD transgenic Alzheimer's disease model group; AD-ASO, 5 × FAD mouse with *Acer truncatum* Bunge seed oil intervention group.

As a major pathological hallmark of AD, Aβ plaques formation commences prominently in 5 × FAD mice between 2 months of age (53). To explore the influence of ASO-administration on Aβ deposition in AD mice, brains were subjected to TS staining. Figure 2A presents representative images of TS staining in specific brain regions. The AD-ASO group showed significantly decreased TS-positive Aβ plaques in various brain regions, comprising dentate gyrus (DG), CA3, CA1 subregions of hippocampus, and the cortex (Figures 2B–I, *P* < 0.01), compared to the AD group. The ELISA results of soluble Aβ1–42 and Aβ1–40 in the brains also indicate that ASO-administration significantly decreased the levels of Aβ protein (Figures 2J, K, *P* < 0.001). The data showed that ASO-intervention effectively decreased Aβ deposition in the brains of 5 × FAD mice.

### ASO administration altered the gut microbiota composition in 5 × FAD mice

ASO reaches the colon, where it undergoes catalysis by the gut microbiota for metabolism. Consequently, it was hypothesized that the potential cognitive enhancement effect of ASO on AD might be attributed to its influence on the composition of the gut microbiota and microbiota-derived metabolites. Microbiota imbalance is regarded as a key pathogenic factor in AD. To test this hypothesis, gut microbiome was conducted by using 16S rRNA gene sequencing of the mouse colon contents.

**Figure 3:**
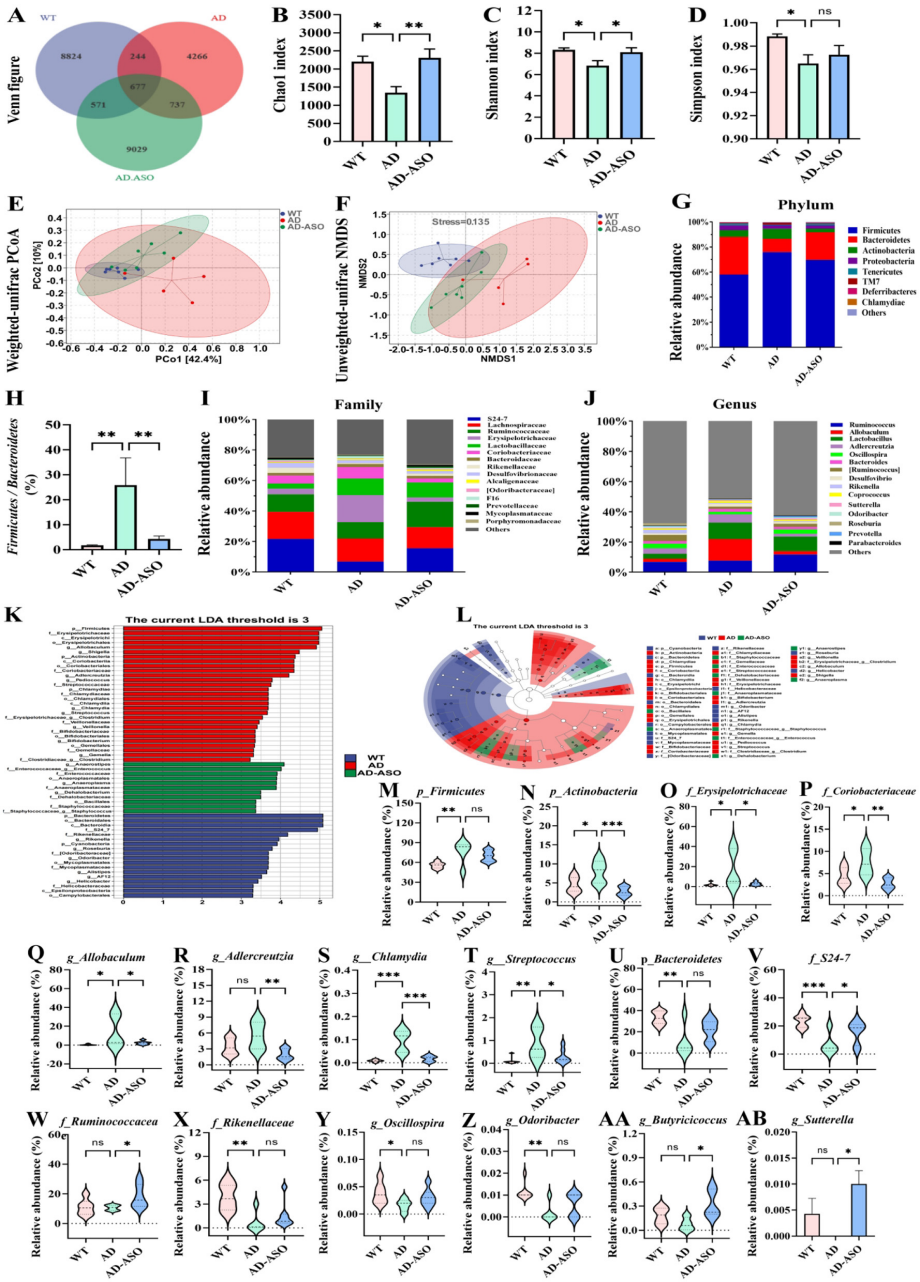
ASO administration reshaped the gut microbiota composition of 5 × FAD mice. **(A)** Venn analysis. **(B)** Chao1 index. **(C)** Shannon index. **(D)** Simpson index. **(E)** Weighted-unifrac PCoA analysis. **(F)** Unweighted-unifrac NMDS analysis. **(G)** Phylum level. **(H)** Ratio of *Firmicutes* to *Bacteriodetes*. **(I)** Family level. **(J)** Genus level. **(K)** LDA score distribution histogram. **(L)** Evolutionary cladogram. The threshold of score of LDA analysis was 3.0. **(M–AB)** Changes in the differential representative bacteria at the phylum, family, and genus levels among three groups. Specifically, the *p_Firmicutes, p_Actinobacteria, f_Erysipelotrichaceae, f_Coriobacteriaceae, g_Allobaculum, g_Adlercreutzia, g_Chlamydia, g_Streptococcus, p_Bacteroidetes, f_S24-7, f_Ruminococcacea, f_Rikenellaceae, g_Oscillospira, g_Odoribacter, g_Butyricicoccus* and *g_Sutterella*. Data are presented as mean ± SEM. *P < 0.05, **P < 0.01, ***P < 0.001. Notable non-significant differences are indicated by “ns” in the figures. WT group *n* = 7; AD group *n* = 5; AD-ASO group *n* = 7; WT, wild-type control group; AD, 5 × FAD transgenic Alzheimer's disease model group; AD-ASO, 5 × FAD mouse with *Acer truncatum* Bunge seed oil intervention group.

Among the WT, AD, and AD-ASO groups of mice, 10,316, 5,924, and 11,014 total OTUs were detected, respectively (Figure 3A). The WT, AD, and AD-ASO groups exhibited 8,824, 4,266, and 9,029 unique OTUs, respectively (Figure 3A). To identify variations in the richness and diversity of gut microbiota, we employed α diversity metrics. A significant decline in the Chao1, Shannon, and Simpson index was observed in AD mice compared with WT mice (Figures 3B–D, *P* < 0.05). ASO-intervention notably increased the Chao1 index and Shannon index (*P* < 0.05), while the Simpson index did not exhibit a significant difference. Additionally, we examined the gut microbiota structure using principal coordinate analysis (PCoA) with weighted_unifrac distance and non-metric multidimensional scaling (NMDS) with unweighted_unifrac distance (Figures 3E, F). Distinct group-specific clustering patterns were evident in both PCoA and NMDS analyses.

Furthermore, we analyzed the structural composition of the gut microbiota. The WT, AD, and AD-ASO groups exhibited notable differences in the composition and quantities of gut microbial species. At the phylum level, three groups primarily comprised *Firmicutes, Bacteroidetes*, and *Actinobacteria* (Figure 3G). The relative abundance of *Firmicutes* and *Actinobacteria* in AD mice was significantly higher than those in WT mice (Figures 3G, M, N, *P* < 0.05), while ASO-supplementation reduced the relative abundance of these phylum in 5 × FAD mice. Conversely, the relative abundance of *Bacteroidetes* displayed an opposite trend (Figures 3G, U). The *Firmicutes* to *Bacteroidetes* ratio showed a significant increase in AD mice compared with WT mice (Figure 3H, *P* < 0.01), while ASO-intervention significantly decreased this ratio (*P* < 0.01). The results indicate that the microbial community composition of the AD-ASO group more closely resembles that of the WT group. The gut microbiota of mice primarily consisted of S24-7, *Ruminococcaceae, Rikenellaceae, Erysipelotrichaceae*, and *Coriobacteriaceae* (Figure 3I), at the family level. Compared to WT mice, the relative abundances of *Erysipelotrichaceae* and *Coriobacteriaceae* were significantly higher in AD mice (Figures 3I, O, P, *P* < 0.05), while ASO-intervention significantly reduced the abundances of *Erysipelotrichaceae* and *Coriobacteriaceae* (*P* < 0.05). The relative abundances of S24-7, *Ruminococcaceae*, and *Rikenellaceae* were lower in AD mice relative to WT mice (Figures 3I, V–X), while ASO-intervention significantly elevated the relative abundance of S24-7 and *Ruminococcaceae* (*P* < 0.05), whereas *Rikenellaceae* exhibited no significant variation.

Microbial taxa showing the greatest differences between groups were identified using the LEfSe method. Using an LDA score threshold of 3.0, the dominant microbial taxa in each group were determined, as illustrated in Figures 3K, L. The results further confirmed that each group's gut microbiota comprised a distinct bacterial community. At the genus level, the gut microbiota composition exhibited substantial variations among different groups (Figure 3J). In comparison with WT mice, AD mice showed significantly elevated relative abundance of *Allobaculum, Adlercreutzia, Chlamydia*, and *Streptococcus* (Figures 3J, Q–T). ASO-intervention significantly reduced the relative levels of these genera (*P* < 0.05). Furthermore, AD mice exhibited decreased relative abundance of *Oscillospira, Odoribacter, Butyricicoccus*, and *Sutterella* compared to WT mice (Figures 3J, Y–AB). Interestingly, ASO-intervention increased the relative abundance of these genera, with significant increases in *Butyricicoccus* and *Sutterella* in 5 × FAD mice (*P* < 0.05). Similarly, ASO-administration significantly increased the abundance of Buty-producing bacteria. ASO markedly increased the abundance of *f_Ruminococcacea, g_Butyricicoccus*, and *g_Sutterella* (Figures 3W, AA, AB, *P* < 0.05), and also increased the abundance of *f_Rikenellaceae, g_Oscillospira*, and *g_Odoribacter*, although not significantly (Figures 3X–Z). These findings demonstrate that ASO-intervention upregulated the generation of short-chain fatty acid (SCFA) and related microbe enrichment in the intestinal of 5 × FAD mice.

The data suggest that ASO-intervention significantly enhanced the growth of gut bacteria correlated with the production of SCFAs, including *f_Ruminococcacea, g_Butyricicoccus, g_Sutterella, f_Rikenellaceae, g_Oscillospira, g_Odoribacter*, and others, particularly those related to gut microbiota-derived Butyrate production.

### ASO administration modified SCFAs in fecal and serum of 5 × FAD mice

**Figure 4:**
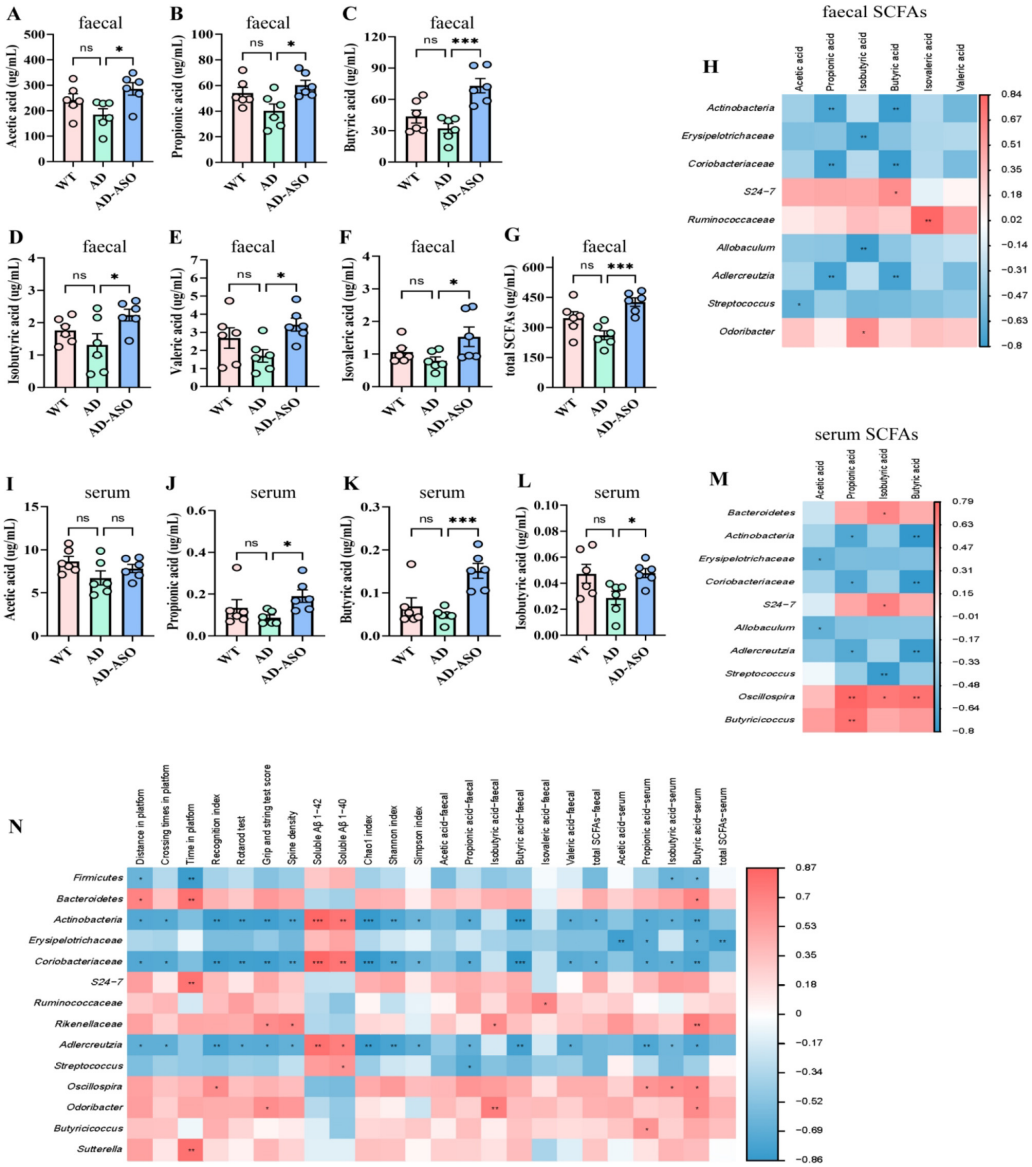
ASO administration upregulated the generation of SCFAs in fresh feces through gut microbiota in 5 × FAD mice. **(A–G)** The concentration of acetic acid, propionic acid, butyric acid, isobutyric acid, valeric acid, isovaleric acid and total SCFAs in feces (μg/ml). **(H)** Heatmap showed Pearson's rank correlated with the relative abundance of gut microbiota and the concentrations of SCFAs in feces of AD mice. **(I–L)** The concentration of acetic acid, propionic acid, butyric acid, and isobutyric acid in serum (μg/ml). Red color: positive correlation; white color: no correlation; and blue color: negative correlation. Significant difference was indicated by asterisks. ^*^P < 0.05, **P < 0.01, ***P < 0.001. **(M)** Heatmap showed Pearson's rank correlated with the relative abundance of gut microbiota and the concentrations of SCFAs in serum of mice. **(N)** Heatmap showed Pearson's rank correlated with the relative abundance of gut microbiota and behavioral tests, and soluble Aβ1–42, soluble Aβ1–40, Chao1, Shannon, and Simpson index, and SCFAs of mice. **(A–G)** and **(I–L)** data are presented as mean ± SEM (*n* = 6). *P < 0.05, **P < 0.01, ***P < 0.001. Notable non-significant differences are indicated by “ns” in the figures. WT, wild-type control group; AD, 5 × FAD transgenic Alzheimer's disease model group; AD-ASO, 5 × FAD mouse *Acer truncatum* Bunge seed oil intervention group.

The SCFAs in mouse feces and serum consist primarily of acetic acid, propionic acid, and butyric acid, among which acetic acid is the most abundant. As shown in Figures 4A–G, the concentrations of acetic acid, propionic acid, butyric acid, isobutyric acid, valeric acid, isovaleric acid and total SCFAs in fresh feces of levels in ASO-administrated mice were markedly higher than those in AD mice (*P* < 0.05), particularly butyric acid. Similarly, the concentrations of propionic acid, butyric acid and isobutyric acid in serum of ASO-administrated mice was markedly higher than those in AD mice (Figures 4I–L, *P* < 0.05). However, the difference of acetic acid is not significant.

To explore the possible link between gut microbiota and the content of SCFAs in feces of mice, data analysis were performed with Pearson's rank correlation. As demonstrated in Figure 4H, the concentration of fecal SCFAs was positively correlated with *S24–7, Ruminococcacea, Odoribacter*, and negatively correlated with *Actinobacteria, Erysipelotrichaceae, Coriobacteriaceae, Allobaculum, Adlercreutzia*, and *Streptococcus*. Similarly, as shown in Figure 4M, the concentration of serum SCFAs was positively correlated with *Bacteriodetes, S24–7, Oscillospira, Butyricicoccus* and negatively correlated with *Actinobacteria, Erysipelotrichaceae, Coriobacteriaceae, Allobaculum, Adlercreutzia*, and *Streptococcus*. These results demonstrate that ASO-administration upregulated SCFA generation and the abundance of related microbe in the intestinal of 5 × FAD mice, particularly those related to Buty production.

Then, relationship between gut microbiota and behavioral indicators and SCFAs were conducted by using Pearson's rank correlation. As illustrated in Figure 4N, the behavioral indicators, SCFAs, and gut microbiota richness and diversity were positively associated with *Bacteriodetes, S24–7, Ruminococcacea, Rikenellaceae, Oscillospira, Odoribacter, Butyricicoccus*, and *Sutterella*, and were negatively correlated with *Fimicutes, Actinobacteria, Erysipelotrichaceae, Coriobacteriaceae, Adlercreutzia*, and *Streptococcus*. These findings indicate that ASO-intervention effectively mitigated AD-induced gut microbiota dysbiosis. Collectively, the results imply that the positive effects of ASO-administration on learning and memory are largely mediated by gut microbiota.

### ASO administration reshaped the metabolic pathways in serum of 5 × FAD mice

**Figure 5:**
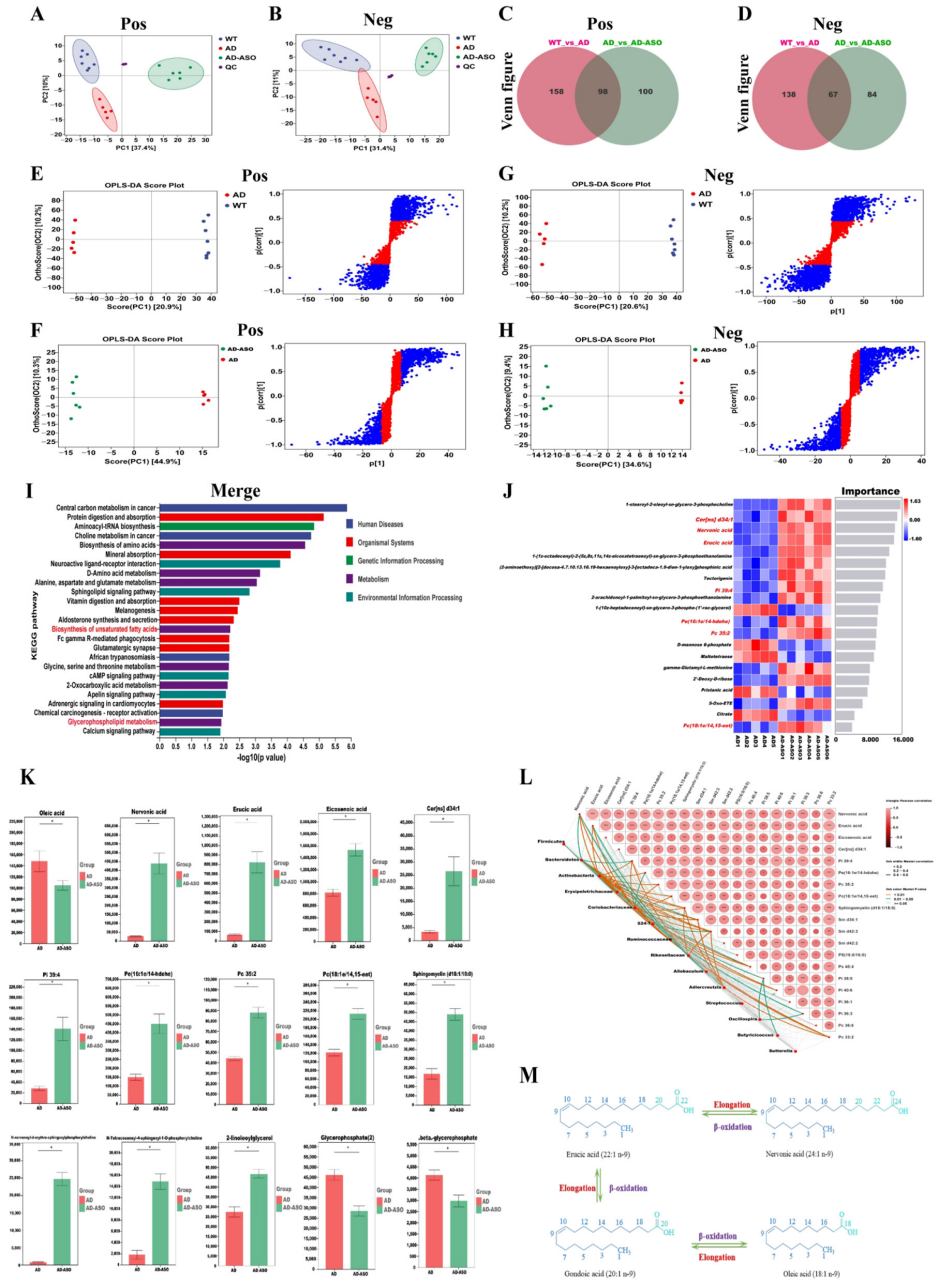
ASO restored the metabolic disruption in 5 × FAD mice. The PCA score plots derived from the serum metabolic profiles in the positive ion mode **(A)** and negative ion mode **(B)**. The Venn figure of the differential metabolites in the positive **(C)** and negative ion modes **(D)**. The OPLS-DA score plots and the corresponding S-plots of serum in the positive **(E, F)** and negative ion mode **(G, H)**. **(I)** AD vs AD-ASO group KEGG enrichment results bar chart in the merge ion mode. **(J)** AD vs AD-ASO group Heatmap of differential metabolites in serum (Top20) in the negative ion mode. **(K)** The differential metabolite expressions level bar chart, about Oleic acid, Nervonic acid, Erucic acid, Eicosenoic acid, Cer[ns] d34:1, Pi 39:4, Pe (16:1e/14-hdohe), Pc 35:2, Pc (18:1e/14, 15-eet), Sphingomyelin (d18:1/18:0), N-nervonoyl-d-erythro-sphingosylphosphorylcholine, N-Tetracosanoyl-4-sphingenyl-1-O-phosphorylcholine, 2-linoleoylglycerol, Glycerophosphate (2), and ß-glycerophosphate, respectively. **(L)** AD vs AD-ASO group Mantel Test visualized the correlation of serum metabolites with gut microbiota. **(M)** Oleic acid, Nervonic acid, Erucic acid, Gondoic acid, after chain elongation or β-oxidation conversion diagram. *P < 0.05. WT group *n* = 7; AD group *n* = 5; AD-ASO group *n* = 6; WT, wild-type control group; AD, 5 × FAD transgenic Alzheimer's disease model group; AD-ASO, 5 × FAD mouse group with *Acer truncatum* Bunge seed oil intervention.

In addition to the dysbiosis of gut microbiota, metabolic change is a significant hallmark of AD. To explore the neuroprotective mechanism of ASO, serum metabolomics was conducted by using untargeted LC/MS/MS. Multivariate statistical analyses were conducted to elucidate detailed metabolic differences among groups. Principal component analysis (PCA) was applied to depict the overall metabolic profile alterations. PCA score plots of serum samples (positive and negative ion modes) are depicted in Figures 5A, B. The tight QC sample clustering confirms the analytical stability. WT and AD groups were clearly separated, reflecting substantial differences in metabolites. After ASO-administration, the metabolite distribution in AD mice approached that of the WT group, demonstrating that ASO-intervention alleviates metabolic disruptions in 5 × FAD mice. Figures 5C, D illustrates the differential metabolites in venn diagrams (positive and negative ion modes). The venn diagram revealed 256 differential metabolites between the WT-vs-AD groups, whereas the AD-vs-AD-ASO groups exhibited 198 differential metabolites in the positive ion mode (Figure 5C), in total 98 differential metabolites shared across the groups. The WT-vs-AD groups had 205 differential metabolites, while the AD-vs-AD-ASO groups had 151 differential metabolites, in the negative ion mode (Figure 5D).

Serum data were subjected to orthogonal projections to latent structures discriminant analysis (OPLS-DA) to elucidate intergroup metabolic differences and pinpoint potential biomarkers. OPLS-DA and S-plots were constructed to differentiate between the WT, AD, and ASO-intervention groups. As illustrated in Figures 5E–H, the OPLS-DA plots of the AD group and each comparative group in both positive and negative ion modes revealed distinct separation. This distinct separation in both ion modes indicates that the established OPLS-DA model possesses robust predictive capability. In the S-plots, compounds represented by blue dots exhibit the highest contribution to group separation. Based on these findings, compounds with a variable importance in projection (VIP) score ≥1 and a *P*-value < 0.05 were designated as potential markers in this study.

KEGG analysis of differential metabolites was used to identify the top 25 pathways with the lowest *P*-values, indicating the most significant enrichment. In the merge ion mode (Figure 5I), the highest-ranked metabolism pathways were the biosynthesis of amino acids, D-amino acid metabolism, alanine, aspartate and glutamate metabolism, biosynthesis of unsaturated FAs, glycine, serine and threonine metabolism, 2-Oxocarboxylic acid metabolism, and glycerophospholipid metabolism, sphingolipid signaling pathway, respectively. The heatmap (Figure 5J) of differential metabolites in serum comparing AD vs AD-ASO groups (Top20) revealed that the top-ranked signature metabolites of inter-group differences were Cer[ns] d34:1, nervonic acid (NA), erucic acid (EA), Pi 39:4, Pe (16:1e/14-hdohe), Pc 35:2, and Pc (18:1e/14, 15-eet), respectively. These results demonstrated that ASO-supplementation significantly increased the concentrations of NA, EA, Eicosenoic acid, Cer[ns] d34:1, Pi 39:4, Pe (16:1e/14-hdohe), Pc 35:2, Pc (18:1e/14, 15-eet), SM (d18:1/18:0), N-nervonoyl-d-erythro-sphingosylphosphorylcholine, N-Tetracosanoyl-4-sphingenyl-1-O-phosphorylcholine, and 2-linoleoylglycerol, respectively (Figure 5K, *P* < 0.05). Conversely, ASO-supplementation significantly decreased the concentrations of oleic acid (OA), glycerophosphate, and β-glycerophosphate, respectively (*P* < 0.05). OA serves as an essential precursor for the biosynthesis of very-long-chain FAs by FA elongase modules. OA and EA can be converted to NA in the brain via elongation of their chain in rats. OA serves as a primary precursor for MUFAs, its reduction may indicate enhanced metabolic flux toward elongation pathways rather than β-oxidation. Given that mitochondrial β-oxidation is significantly impaired in the AD brain, fatty acids may be preferentially redirected from energy metabolism toward structural lipid synthesis. EA, as an intermediate very-long-chain MUFAs, may therefore accumulate due to limited downstream oxidation capacity, explaining why its level exceeds that of NA. Multiple studies have demonstrated NA deficiency in individuals with neurodegeneration, and NA supplementation as a type of nutraceutical is considered to improve brain develop-ment and cognition. Importantly, NA is a critical component of neuronal membranes and myelin. Its selective elevation, despite lower abundance relative to EA, may reflect functional demand-driven synthesis or retention to support neuronal membrane integrity and synaptic remodeling under neurodegenerative conditions. Together, these findings suggest that ASO induces adaptive modulation of ω-9 fatty acid metabolism in the context of mitochondrial dysfunction, which may contribute to AD amelioration. Consequently, after ASO-supplementation, the concentration of OA decreased, while the concentrations of NA, EA, and Eicosenoic acid increased. Figure 5M illustrates the chain elongation or β-oxidation conversion diagram for OA, NA, EA, and Gondoic acid (also known as 11-Eicosenoic acid). These findings indicate that ASO-supplementation primarily reshaped the biosynthesis of unsaturated FAs, glycerophospholipid metabolism, and sphingolipids metabolism.

We subsequently performed a Mantel Test to examine the relationship between differential serum metabolites, primarily associated with lipid metabolism, and the differential bacterial genera identified through LEfSe analysis. The results demonstrated a significant correlation between alterations in serum metabolites and changes in bacterial genera (Figure 5L). The results indicate that gut microbiota dysbiosis in AD mice contributes to metabolic disturbances, while ASO-intervention may help restore microbial balance, thereby alleviating these metabolic disruptions. This mechanism could potentially explain the neuroprotective effects of ASO observed in AD mice.

### ASO administration altered the phospholipid metabolism-related signaling pathways 5 × FAD mice

**Figure 6:**
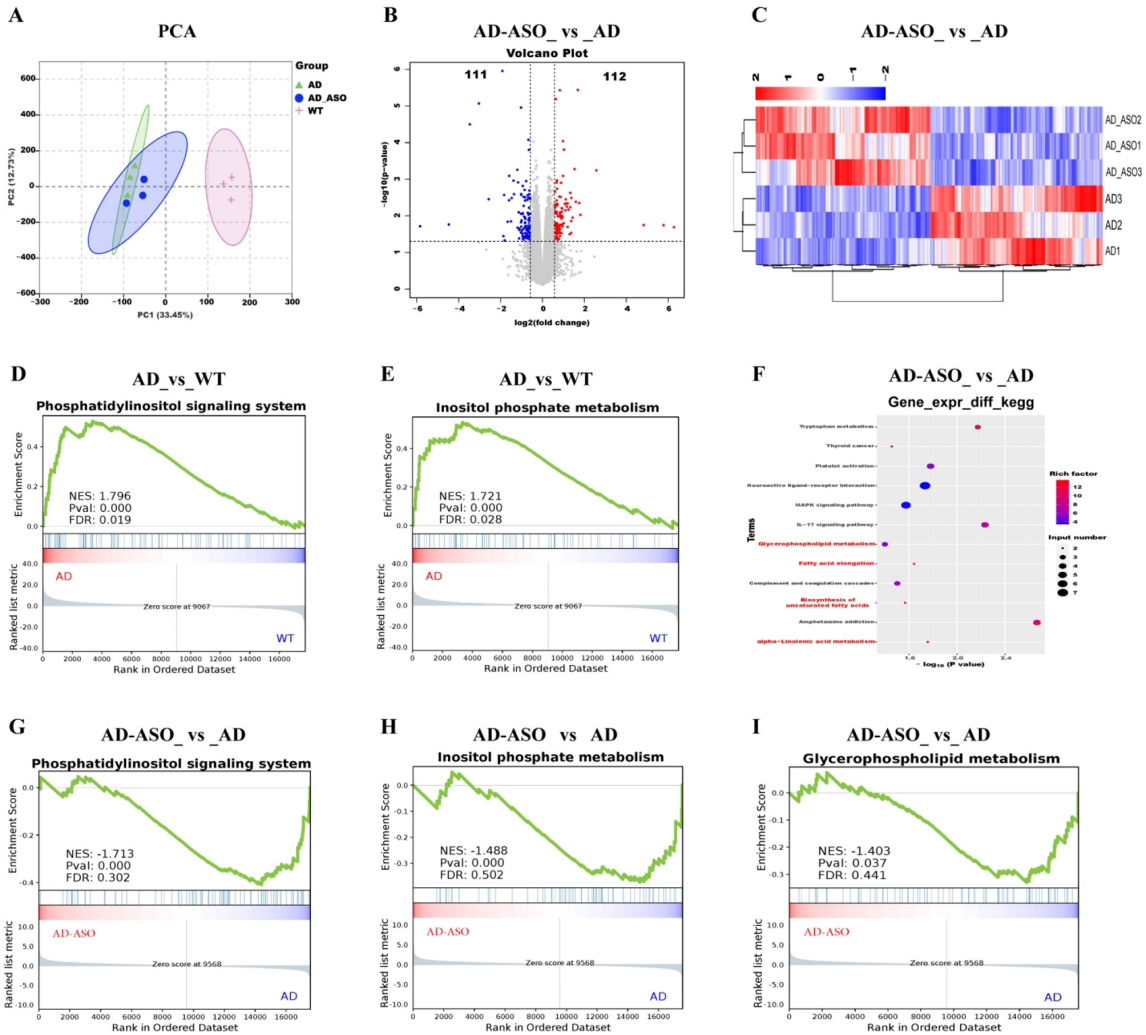
ASO-administration altered the phospholipid metabolism-related signaling pathways in 5 × FAD mice. **(A)** PCA diagrams. **(B)** AD-ASO_ vs _AD group volcano plot. **(C)** AD-ASO_ vs _AD group clustering heatmap of differentially expressed mRNA genes. **(D, E)** AD_vs_WT group GSEA in transcriptomics shows the enrichment of phosphatidylinositol signaling system and inositol phosphate metabolism pathway gene sets. **(F)** KEGG enrichment analysis revealed pathways that were upregulated and downregulated in AD-ASO mice in comparison with the AD group. **(G–I)** AD-ASO_ vs_ AD group GSEA in transcriptomics shows the enrichment of phosphatidylinositol signaling system, inositol phosphate metabolism and glycerophospholipid metabolism pathway gene sets. WT, AD and AD-ASO group *n* = 3; WT, wild-type control group; AD, 5 × FAD transgenic Alzheimer's disease model group; AD-ASO, 5 × FAD mouse *Acer truncatum* Bunge seed oil intervention group.

RNA-sequencing of the hippocampus was performed to elucidate the signaling pathways associated with ASO-mediated effects. Figure 6A displays PCA plots. The observed separation between the WT and AD groups highlights marked differences in mRNA expression. Figure 6B shows volcano plot of mRNA expression, while gray dots denote non-differentially expressed genes, blue dots indicate significantly downregulated, and red dots denote significantly upregulated. The analysis reveals that 112 genes were upregulated and 111 genes downregulated in AD-ASO group relative to the AD group. Additionally, the hierarchical clustering heatmap illustrates the differentially expression patterns of mRNA between the AD-ASO and AD groups (Figure 6C).

Differentially expressed genes are typically identified based on two criteria: fold change and significance level. Here, genes are defined as differentially expressed if they meet the threshold of |logFC| >0.58 and *P*-value < 0.05. Enrichment analysis was conducted by using KEGG pathways as units to identify pathways significantly enriched among the differentially regulated genes. Subsequently, GSEA compares gene expression data with known gene sets. By analyzing gene expression profiles, it helps to understand the enrichment of these genes in known functional gene sets, explores their association with phenotypes, and determines whether their expression levels show statistical significance. This analysis uses pyGSEA, with background datasets from GO (2018) and KEGG (2019). Figures 6D, E GSEA results indicated that in AD group the expression of phosphatidylinositol signaling system and inositol phosphate metabolism gene sets was upregulated compared to those in WT group. The highest-ranked enriched KEGG pathways, ranked by *P*-value, KEGG enrichment analysis revealed that the significant altered pathways after ASO-intervention in AD mice were primarily glycerophospholipid metabolism, FA elongation, biosynthesis of unsaturated FAs, and α-linolenic acid (ALA) metabolism, respectively (Figure 6F). Figures 6G–I GSEA results showed that AD-ASO group demonstrated down-regulation of phosphatidylinositol signaling system, inositol phosphate metabolism, and glycerophospholipid metabolism gene sets compared to the AD group. The data suggest that in the AD group phospholipid metabolism-related signaling pathways was up-regulated relative to the WT group, while in the AD-ASO group this signaling pathway was down-regulated relative to the AD group. Importantly, AD mice exhibited abnormal phospholipid metabolism, while ASO-intervention was shown to restored phospholipid metabolism balance. This suggests that ASO-intervention reshapes phospholipid metabolism in brain of 5 × FAD mice, thereby potentially providing neuroprotective effects.

### ASO administration mitigated neuroinflammation, decreased systemic inflammation, and enhanced antioxidant stress in 5 × FAD mice

**Figure 7:**
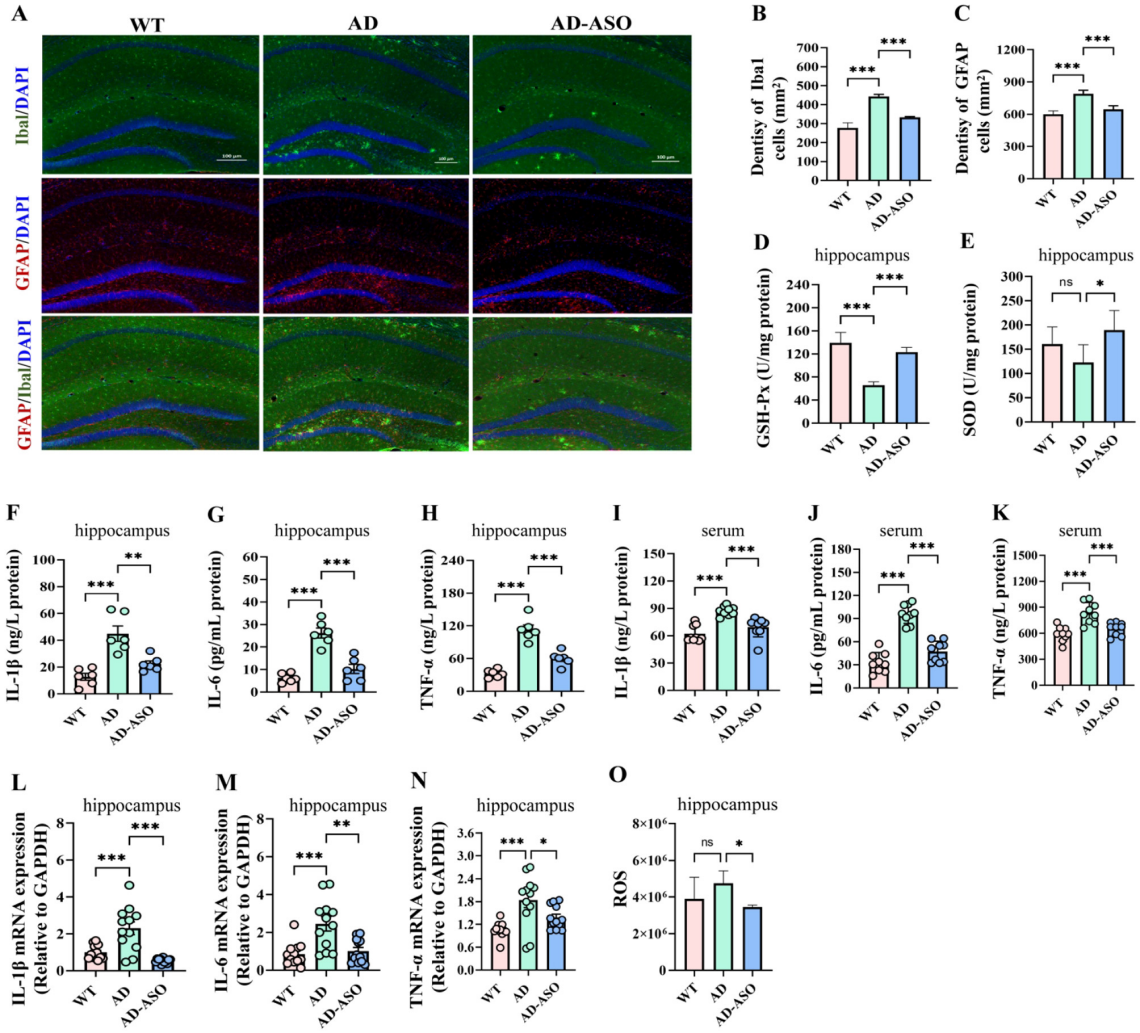
ASO mitigated neuroinflammatory response, decreased systemic inflammation, and enhanced oxidative stress in 5 × FAD mice. **(A)** Representative images of Iba1 and GFAP immunofluorescence staining in the hippocampus. **(B, C)** Quantification of Iba1 and GFAP are based on immunofluorescence staining sections by ImageJ software (*n* = 6 slices from three mice). **(D, E)** The levels of GSH-Px and SOD in the hippocampus (*n* = 6). **(F–H)** The levels of IL-1β, IL-6, and TNF-α in the hippocampus by ELISA kits (*n* = 6); **(I–K)** The levels of IL-1β, IL-6, and TNF-α in the serum by ELISA kits (*n* = 6); **(L–N)** The mRNA expression levels of IL-1β, IL-6, and TNF-α in the hippocampus by RT-PCR (*n* = 6). **(O)** The levels of ROS in the hippocampus (*n* = 6). Data are presented as mean ± SEM. *P < 0.05, **P < 0.01, ***P < 0.001. WT, wild-type control group; AD, 5 × FAD transgenic Alzheimer's disease model group; AD-ASO, 5 × FAD mouse with *Acer truncatum* Bunge seed oil intervention group.

Neuroinflammation is a major factor in AD progression. Patients with AD experience show disruption in the homeostatic function of microglia and astrocytes, resulting in a neuroinflammatory environment in the brain. This inflammatory response within the CNS encompasses immune cell infiltration, microglial and astrocyte activation, and pro-inflammatory cytokine release. One key feature of neuroinflammation in AD is the accumulation of activated microglia around Aβ plaques. Astrocytes, the main glial cell type in the CNS, are considered key players in neurodegenerative diseases, involved in synaptic function, synaptic loss, and neuronal death. To investigate the impact of ASO on microglia and astrocytes related to neuroinflammation in 5 × FAD mice, we labeled microglia and astrocytes in the hippocampus using Iba1 for microglia and GFAP for astrocytes, as shown in Figure 7A. Compared to WT mice, AD mice showed a significantly increased in hippocampal Iba1-positive cells (Figure 7B, *P* < 0.001). Interestingly, ASO-administration significantly reduced the density of Iba1-positive cells in AD mice (*P* < 0.001), indicating that ASO could reduce microglial proliferation. Similar trend was detected in the density of GFAP-positive cells (Figure 7C, *P* < 0.001). These findings suggest enhanced proliferation and increased density of microglia and astrocytes in 5 × FAD mice, implying the presence of neuroinflammation. Importantly, ASO-administration significantly inhibited the proliferation of microglia and astrocytes in AD mice.

Previous research has demonstrated that excessive neuroinflammation promotes oxidative responses, which subsequently exacerbate inflammation. GSH-Px and SOD are crucial antioxidant enzymes in the body, and their activity reflects the body's antioxidant capacity. In this study, hippocampal GSH-Px was significantly decreased in AD mice relative to WT mice (Figure 7D, *P* < 0.001). Interestingly, ASO-administration increased GSH-Px levels in AD mice (*P* < 0.001). Additionally, ASO-administration elevated SOD levels in the hippocampus of AD mice (Figure 7E, *P* < 0.05). On the contrary, ASO-administration significantly decreased the ROS levels of AD mice hippocampus (Figure 7O, *P* < 0.05). Collectively, these data incate that ASO-intervention enhanced antioxidant function in 5 × FAD mice.

In comparison to those in WT mice, ELISA analysis revealed that hippocampal levels of pro-inflammatory cytokines such as IL-1β, IL-6, and TNF-α were markedly increased in AD mice (Figures 7F–H, *P* < 0.001), while ASO-intervention significantly decreased the levels of these cytokines (*P* < 0.01). ELISA experiments revealed similar trend of IL-1β, IL-6, and TNF-α in serum (Figures 7I–K, *P* < 0.001). Consistently, the mRNA of pro-inflammatory cytokines in the hippocampus exhibited the same pattern (Figures 7L–N, *P* < 0.05). The results showed that the IL-1β, IL-6, and TNF-α, which are closely related to inflammation, are elevated in 5 × FAD mice, and ASO-intervention can significantly inhibit the expression of these cytokines in AD mice. Collectively, the results demonstrate that ASO-supplementation effectively mitigated neuroinflammation, reduced systemic inflammation, and enhanced antioxidant stress in the 5 × FAD mice.

### FMT reduced Aβ deposition, alleviated learning and memory dysfunction and neuroinflammation in 5 × FAD mice

**Figure 8:**
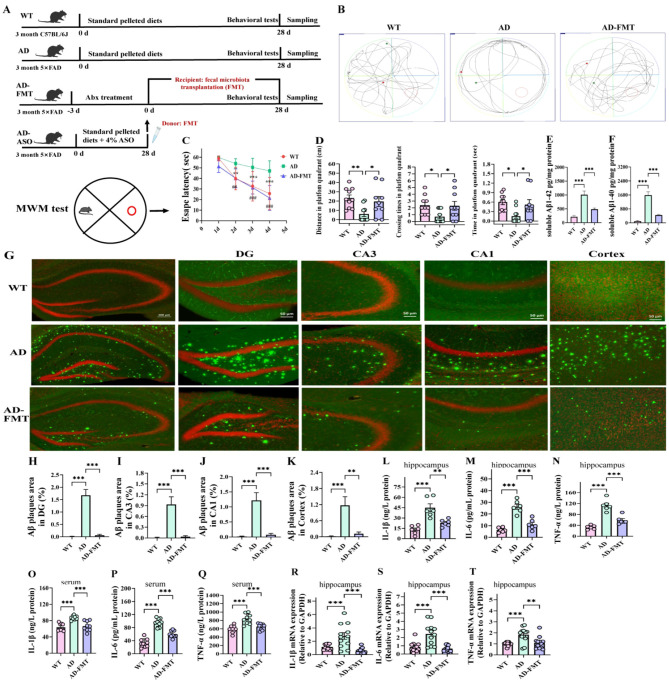
FMT alleviated learning and memory deficits Aβ deposition and neuroinflammatory response in 5 × FAD mice. **(A)** The experiment workflow of animal-administration. **(B)** Representative track images of mice in the MWM test. **(C)** Escape latency during training days in MWM test (*n* = 10), **P < 0.01, ***P < 0.001, WT group compared to the AD group; ^##^*P* < 0.01, ^###^*P* < 0.001, AD-FMT group compared to the AD group; Data are presented as mean ± SEM, and statistical significance was determined by Two-way ANOVA with Tukey's test. **(D)** Distance in platfom quadrant, crossing times in platfom quadrant, time in platfom quadrant *n* = 10. **(E, F)** Soluble Aβ1–42 and Aβ1–40 content in mouse brain (*n* = 6). **(G)** Representative images of TS staining in indicated brain regions. **(H–K)** Quantification of the Aβ plaques (*n* = 9 brain sections, from three mice per group) in the hippocampus DG, CA3, and CA1 subregions, and the cortex from indicated group mice. **(L–N)** The levels of IL-1β, IL-6, and TNF-α in the hippocampus by ELISA kits (*n* = 6); **(O–Q)** The levels of IL-1β, IL-6, and TNF-α in the serum by ELISA kits (*n* = 6); **(R–T)** The mRNA expression levels of IL-1β, IL-6, and TNF-α in the hippocampus (*n* = 6). Data are presented as mean ± SEM. *P < 0.05, **P < 0.01, ***P < 0.001. WT, wild-type control group; AD, 5 × FAD transgenic Alzheimer's disease model group; AD-FMT, 5 × FAD transgenic Alzheimer's disease mouse with fecal microbiota transplantation group.

To examine the causal involvement of gut microbiota in ASO amelioration of cognitive dysfunction and Aβ accumulation in 5 × FAD mice, we transplanted the gut microbiota from ASO-supplemented AD mice (AD-ASO group) to AD mice (AD-FMT group). To improve FMT efficacy, mice were pretreated with ABx for 3 days to deplete resident gut microbiota. Figure 8A illustrates the experimental workflow of administration. To assess the ameliorative effect of FMT on memory impairment in male 5 × FAD mice, MWM tests were conducted after daily FMT for 28 days. In the navigation test (Figure 8C), relative to the WT group, the AD group exhibited longer escape latency in a circular motion to find the platform on days 2–4 (Figure 8C, *P* < 0.01). Interestingly, compared to the AD group, AD-FMT mice demonstrated lower escape latency to find the platform on days 2–4 (*P* < 0.01). Furthermore, the spatial probe test on day 5 (Figure 8B) demonstrated that AD-FMT mice had significantly longer distance, larger number of cross-platforms, and more time in the target quadrant relative to the AD mice (Figure 8D, *P* < 0.05). The findings show that FMT enhanced the learning and memory abilities of AD mice.

Subsequently, to explore the impact of FMT on Aβ accumulation in 5 × FAD mice, we conducted TS staining (Figure 8G). The findings revealed that, relative to the AD mice, the FMT mice showed significantly reduced TS-positive Aβ plaques in various brain regions, including the hippocampal DG, CA3, and CA1 subregions, as well as the cortex (Figures 8H–K, *P* < 0.01). ELISA results of soluble Aβ1–42 and Aβ1–40 in the brains of AD mice also indicate that FMT inhibited the levels of Aβ protein (Figures 8E, F, *P* < 0.001), relative to the AD group. The findings indicate that FMT significantly reduced Aβ accumulation in the brains of 5 × FAD mice.

Additionally, we analyzed the expression of IL-1β, IL-6, and TNF-α through ELISA and qPCR techniques. AD mice exhibited significantly increased levels of IL-1β, IL-6, and TNF-α in the hippocampus relative to WT mice (Figures 8L–N, R–T, *P* < 0.001). However, FMT substantially decreased the levels of these cytokines (*P* < 0.01). A comparable trend was noted in the serum levels of these cytokines (Figures 8O–Q, *P* < 0.001). These findings demonstrate that FMT effectively mitigated neuroinflammation and decreased systemic inflammation in the 5 × FAD mice.

### FMT enhanced the abundance of SCFAs-producing bacteria and reshaped serum metabolism in 5 × FAD mice

**Figure 9:**
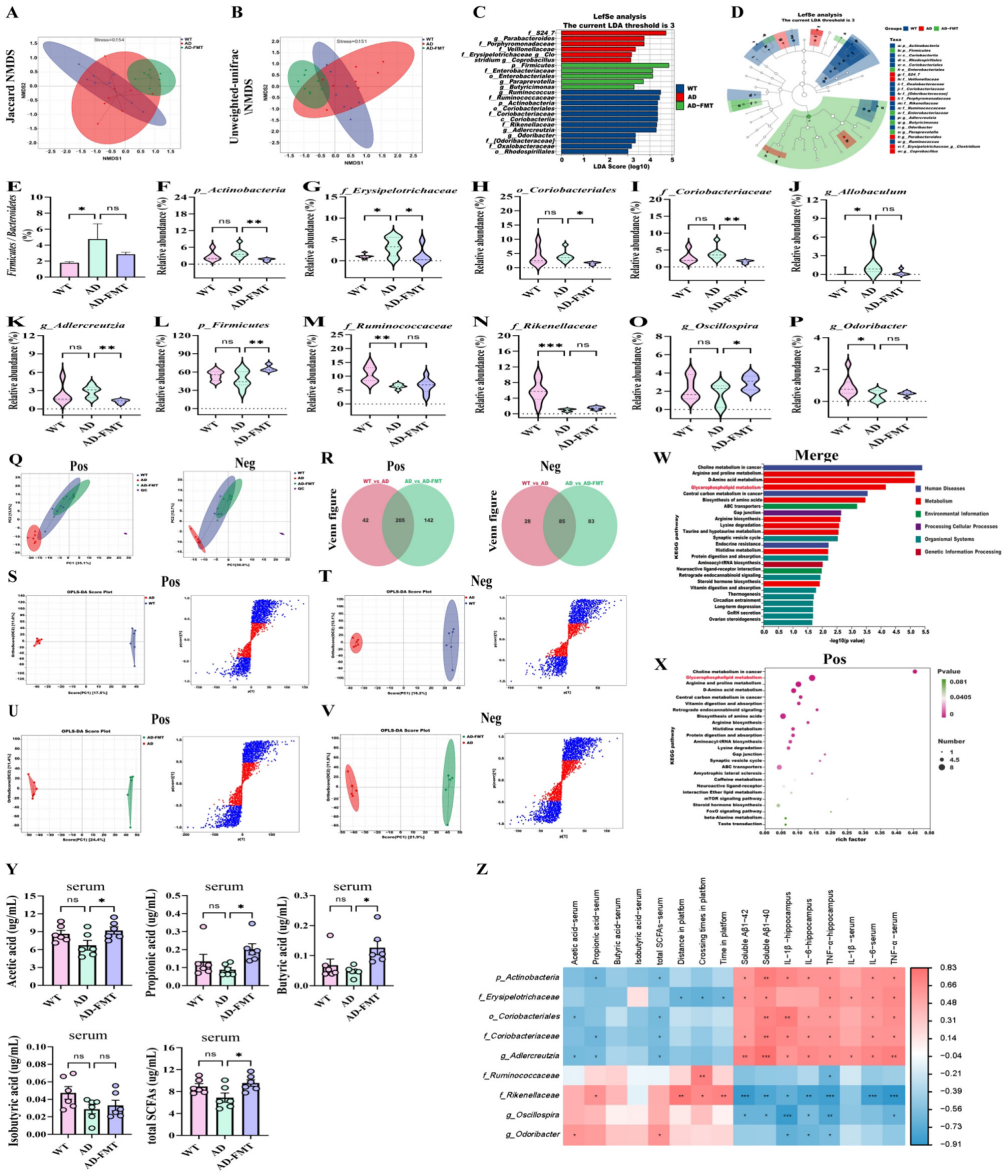
FMT regulated the composition of gut microbiota, and altered differential metabolites in serum of 5 × FAD mice. **(A)** Jaccard NMDS analysis. **(B)** Unweighted-unifrac NMDS Analysis. **(C)** LDA score distribution histogram. **(D)** Evolutionary cladogram. The threshold of score of LDA analysis was 3.0. **(E)** Ratio of *Firmicutes* to *Bacteriodetes* (*n* = 7). **(F–P)** Changes in the differential representative bacteria at the phylum, order, family, and genus levels among the WT, AD, and AD-FMT groups (*n* = 7). Specifically, the *p_Actinobacteria, f_Erysipelotrichaceae, o_Coriobacteriales, f_Coriobacteriaceae, g_Allobaculum, g_Adlercreutzia, p_Firmicutes, f_Ruminococcaceae, f_Rikenellaceae, g_Oscillospira* and *g_Odoribacter*, respectively. **(Q)** The PCA score plots derived from the serum metabolic profiles in the positive ion mode and negative ion mode. **(R)** The Venn figure of the differential metabolites in the positive and negative ion modes. The OPLS-DA score plots and the corresponding S-plots of serum in the positive **(S–U)** and negative ion mode **(T–V)**. The AD vs AD-ASO group KEGG enrichment results bar chart in the merge ion mode **(W)** and positive ion mode **(X)**. **(Q–X)** *n* = 6. **(Y)** The concentrations of acetic acid, propionic acid, butyric acid, isobutyric acid and total SCFAs in serum (μg/ml). **(Z)** Heatmap showed Pearson's rank correlation between the relative abundance of gut microbiota and SCFAs in serum, and MWM tests, and soluble Aβ1–42, soluble Aβ1–40, in addition the levels of IL-1β, IL-6, and TNF-α in the hippocampus and serum. Red color: positive correlation; white color: no correlation; and blue color: negative correlation. Signifificant difffference was indicated by asterisks. ^*^P < 0.05, **P < 0.01, ***P < 0.001. Data are presented as mean ± SEM. *P < 0.05, **P < 0.01, ***P < 0.001. WT, wild-type control group; AD, 5 × FAD transgenic Alzheimer's disease model group; AD-FMT, 5 × FAD transgenic Alzheimer's disease mouse with fecal microbiota transplantation group.

Following 28 days of daily FMT, we investigated shifts in the gut microbial community structure of AD mice. Jaccard and unweighted-unifrac NMDS analyses (Figures 9A, B) revealed that the gut microbiota composition and structure of AD-FMT mice resembled that of WT mice. Subsequently, LEfSe analysis (Figures 9C, D) with an LDA score threshold of 3.0 identified high-abundance microbes in this group. Figure 9E depicts the *Firmicutes* to *Bacteroidetes* ratio. These results further indicated that each group's gut microbiota comprised a specific bacterial community. Specifically, the relative abundances of *p_Actinobacteria, f_Erysipelotrichaceae, o_Coriobacteriales, f_Coriobacteriaceae*, and *g_Adlercreutzia* decreased in AD-FMT mice relative to the AD mice (Figures 9F–I, K, *P* < 0.05), respectively. The *g_Allobaculum* showed similar trend, although without statistical significance (Figure 9J). Concurrently, the relative abundances of *p_Firmicutes, f_Ruminococcaceae, f_Rikenellaceae, g_Oscillospira*, and *g_Odoribacter* elevated in AD-FMT mice relative to AD mice (Figures 9L–P), respectively. FMT significantly increased the abundance of *p_Firmicutes* and *g_Oscillospira* (Figures 9L, O, *P* < 0.05). These findings demonstrate that FMT modulated the structure and composition of the gut microbiota.

Afterward, serum metabolic analysis was performed to investigate the neuroprotective mechanism of FMT. The PCA analysis revealed distinct separation between the WT and AD groups, showing marked metabolic differences (under positive and negative ion modes; Figure 9Q). The metabolic distribution of AD-FMT mice was closer to the WT mice, suggesting that FMT could mitigate metabolic disruption in 5 × FAD mice. The Venn diagram (Figure 9R) showed 247 differential metabolites between the WT-vs-AD groups, while the AD-vs-AD-FMT groups exhibited 347 differential metabolites in the positive ion mode. The WT-vs-AD and AD-vs-AD-FMT groups shared 205 differential metabolites. The WT-vs-AD groups had 113 differential metabolites, whereas the AD-vs-AD-FMT groups had 168, in the negative ion mode. The OPLS-DA analysis (Figures 9S–V) demonstrated clear separation between the AD group and other groups in both positive and negative ion modes. Based on these findings, potential markers were defined as compounds with VIP ≥1 and *P*-value < 0.05.

KEGG analysis of differential metabolites was used to identify the top 25 pathways with the lowest *P*-values, indicating the most significant enrichment. Figure 9W, representing the merged ion mode, shows that the top-ranked metabolism pathways were Arginine and proline metabolism, D-amino acid metabolism, and Glycerophospholipid metabolism, respectively. The top-ranked metabolism pathways were Glycerophospholipid metabolism, Arginine and proline metabolism, and D-amino acid metabolism, respectively, in the positive ion mode (Figure 9X). The findings indicated that FMT primarily affected the glycerophospholipid metabolism pathway.

Next, we examined the concentration of SCFAs in serum. As depicted in Figure 9Y, acetic acid, propionic acid, butyric acid, and total SCFAs in AD-FMT mice showed significantly higher concentrations relative to the AD mice (Figure 9Y, *P* < 0.05), and the difference of isobutyric acid is not significant. Pearson's rank correlation was applied to assess the possible relationships between gut microbiota and other physiological indicators in AD mice. Findings indicated that the concentration of SCFAs was positively associated with the abundance of *f_Ruminococcacea, f_Rikenellaceae, g_Oscillospira, g_Odoribacter*, and negatively associated with the abundance of *p_Actinobacteria, f_Erysipelotrichaceae, o_Coriobacteriales, f_Coriobacteriaceae* and *g_Adlercreutzia* (Figure 9Z). The soluble Aβ1–42, Aβ1–40, in addition to IL-1β, IL-6, and TNF-α levels in the hippocampus and serum detected by ELISA kits showed opposing trend. Our data indicate that the positive effects of ASO-intervention on learning and memory are largely dependent on gut microbiota and its metabolites.

### Supplementation of sodium Buty mitigated learning and memory impairments in 5 × FAD mice

**Figure 10:**
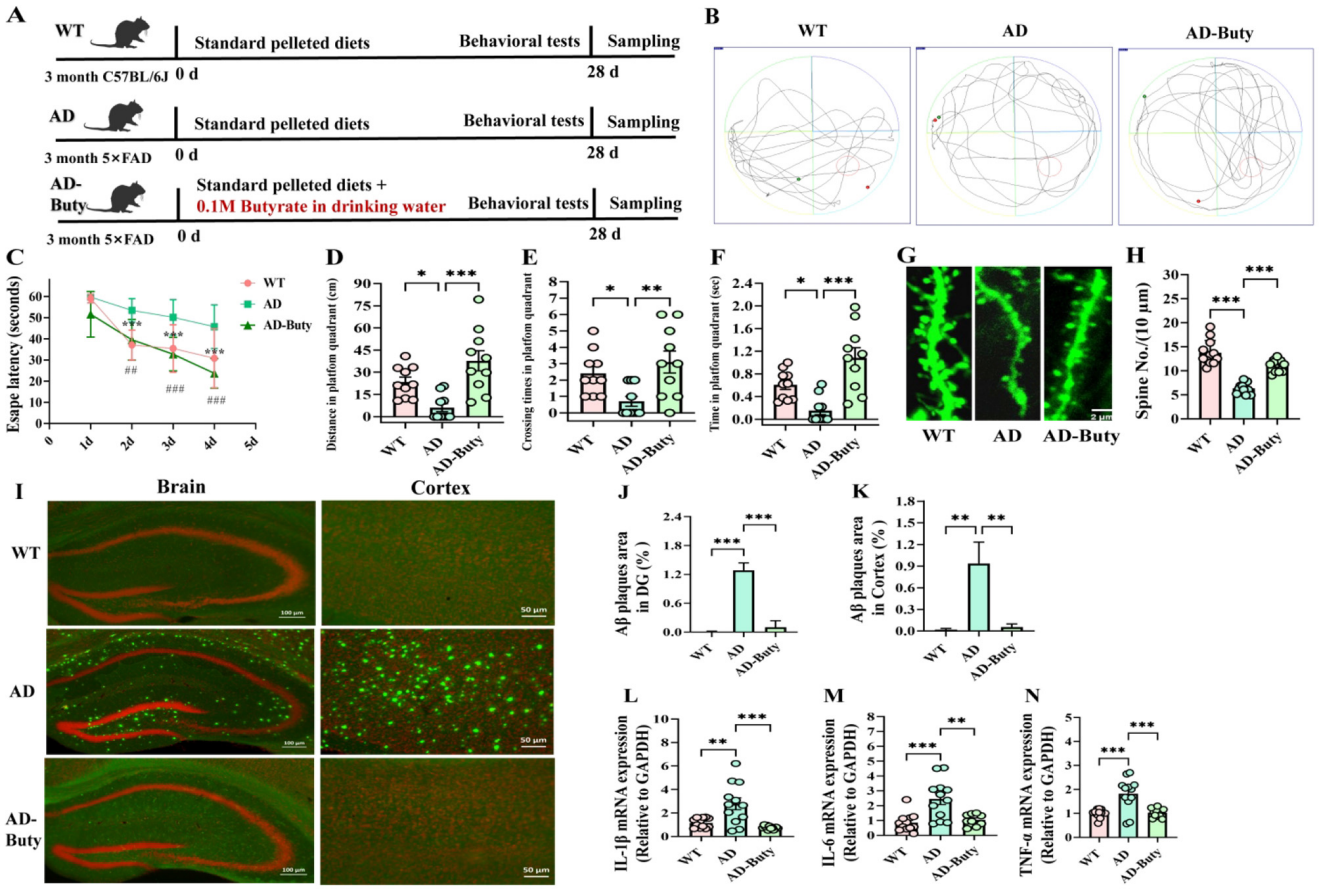
Sodium buty mitigated learning and memory impairment in 5 × FAD mice. **(A)** The experiment workflow of animal-administration. **(B)** Representative track images of mice in the MWM test. **(C)** Escape latency during training days in MWM test (*n* = 10, ***P < 0.001, WT group compared to the AD group; ^##^*P* < 0.01, ^###^*P* < 0.001, AD-Buty group compared to the AD group) Data are presented as mean ± SEM, and statistical significance was determined by Two-way ANOVA with Tukey's test. **(D)** Distance in platform quadrant. **(E)** Crossing times in platform quadrant. **(F)** Time in platform quadrant. **D–F** (*n* = 10). **(G)** Representative images of tertiary dendritic segments and spines of granule cells in the dentate gyrus of YFP mice (*n* = 9 brain sections, from three mice per group). **(H)** Dendritic spine density in the hippocampus measured by Image J. **(I)** Representative images of TS staining in indicated brain regions (*n* = 9 brain sections, from three mice per group). **(J-K)** Quantification of the Aβ plaques in the hippocampus DG regions, and the cortex from indicated group mice (*n* = 3). **(L-N)** The mRNA levels of IL-1β, IL-6, and TNF-α in the hippocampus (*n* = 6). Data are presented as means ± SEM. *P < 0.05, **P < 0.01, ***P < 0.001. WT, wild-type control group; AD, 5 × FAD transgenic Alzheimer's disease model group; AD-Buty, 5 × FAD mouse with sodium Butyrate intervention group.

The elevated presence of SCFA-producing microorganisms, especially Buty-producing bacteria in the intestinal microbiome, represents the primary mechanism by which ASO mitigates memory deficits in 5 × FAD mice. Buty, a microbiota-derived metabolite. To strengthen the validation of of Buty's effects in AD mice, we supplemented their drinking water with 0.1 M Buty for a period of 28 days (Figure 10A). Representative tracking images of mice in the MWM test (Figure 10B). In the navigation test (Figure 10C), the AD mice exhibited significantly longer escape latency in a circular motion to locate the platform on days 2–4 relative to the WT mice (Figure 10C, *P* < 0.001). Conversely, AD-Buty mice demonstrated reduced escape latency to find the platform on days 2–4 relative to the AD mice (*P* < 0.01). Furthermore, in the probe tests on day 5 (Figure 10B), AD-Buty mice showed significantly increased distance, higher number, and extended time in the target quadrant relative to the AD mice (Figures 10D–F, *P* < 0.01). Figure 10G illustrates the tertiary dendritic segments of granule cells in the DG of YFP mice. Sodium Buty supplementation significantly increased dendritic spine density in mice with AD (Figures 10G, H, *P* < 0.001). The results show that sodium Buty alleviates learning and memory impairments in 5 × FAD mice by promoting the formation of dendritic spines.

Figure 10I presents representative TS-stained images in specific brain regions. The findings demonstrate that AD-Buty mice showed a significantly reduction in TS-positive Aβ plaques in the hippocampal DG and cortex, relative to the AD mice (Figures 10J, K, *P* < 0.01). The results suggest that sodium Buty supplementation substantially decreased Aβ accumulation in the brains of 5 × FAD mice. In comparison to the WT mice, the AD mice exhibited significantly elevated mRNA expression of IL-1β, IL-6, and TNF-α in the hippocampus (Figures 10L–N, *P* < 0.01). However, sodium Buty intervention substantially reduced these pro-inflammatory cytokine levels (*P* < 0.01).

## Discussions

AD is a neurodegenerative disorder characterized by deficits in learning and memory, ultimately affecting speech, behavior, visual-spatial localization, and motor function. Animal behavioral experiments serve as a crucial tool in contemporary neuroscience research, enabling comprehensive assessment of various behavioral aspects in mice, including learning and memory, cognitive function, motor coordination, endurance, and emotional states. In this research, the MWM, NOR, rotarod, and string tests showed that ASO-administration effectively mitigated impairment of learning and memory while improving motor coordination and endurance in 5 × FAD mice.

Currently, AD treatments primarily focus on symptom alleviation rather than addressing root causes. However, evidence suggests that clearing Aβ oligomers and plaques with monoclonal antibodies can decelerate AD progression (54). Notably, lecanemab, a monoclonal antibody targeting aggregated Aβ, has demonstrated efficacy in slowing disease progression in a rigorous, multicenter, randomized, double-blind phase three clinical trial. This landmark advancement establishes AD as a modifiable condition, with abnormal Aβ aggregation recognized as a key etiological factor (55). Presently, clinical treatments for AD and other neurodegenerative disorders often target a single molecular pathway. However, due to the complex pathogenesis of these diseases, therapeutic outcomes remain suboptimal. In contrast, natural products have attracted growing interest owing to their multi-target effects and relative safety, making them promising candidates. Therefore, identifying effective natural products or functional foods for AD prevention and treatment represents a crucial scientific challenge that warrants further investigation.

The plant-derived ASO, rich in unsaturated FAs such as LA, OA, and NA, is highly valued for its exceptional nutritional and therapeutic benefits. *Acer truncatum* has emerged as a novel source of NA. ASO is abundant in NA, and studies have demonstrated its high concentration in brain, especially the white matter, where the myelinated nerve fibers are the major components. Deficiency in NA leads to brain disorders such as autism, AD, brain atrophy, and memory loss. As a key component of membranes in neurons and myelin sheath, NA has attracted growing interest due to its potential to promote the regeneration of damaged, diseased, or dormant neurons and myelin sheath, positioning it as a potential candidate for future applications. NA, classified as a very long-chain FA, is essential for human health, particularly contributing to neural development and repair. It is a major component of lipid molecules such as PC, SM, and ceramide, in which it contributes to cell membrane integrity and function in the nervous system (56, 57). Furthermore, NA is a primary constituent of sphingolipids within the myelin sheath and plays a vital role in supporting brain development and the integrity of peripheral nervous tissue (58). EA and OA can be converted to NA in the brain via elongation of their chain in rats (59, 60). OA is formed through beta-oxidation of EA. OA functions as a key precursor for the biosynthesis of very-long-chain FAs *via* the FA elongase module. FAs are key nutrients necessary for human growth and bodily functions, with their composition closely linked to brain health. This underscores its significance as a vital structural lipid. Additionally, LA can protect neurons via its antioxidant and anti-inflammatory activities, which may help mitigate AD (61). ALA, an unsaturated FA, is vital for nervous system health. ALA protects neurons by inhibiting Aβ-related toxicity and apoptosis, and by reducing pathological tau aggregation (62). Dietary supplements containing PUFA ameliorates cognitive deficits and improves hippocampal synaptic plasticity in AD animal models. Our recent studies have found that dietary supplementation with the natural product ASO in mice promotes myelin regeneration and prevents the course of multiple sclerosis by promoting the maturation of oligodendrocytes. Accordingly, we carried out this study to explore the potential preventive and therapeutic roles of ASO in AD.

Recent studies suggest that early AD is closely associated with the pathological buildup of Aβ and subsequent plaques formation (63). During the initial phase of AD, abnormal Aβ accumulation activates microglia, which then migrate to plaque sites to phagocytose and degrade Aβ deposits. As the disease progresses and Aβ accumulation intensifies, microglial clearance becomes insufficient, resulting in the buildup of Aβ plaques. This worsening pathology further over-activates microglia, amplifying the inflammatory response throughout the brain. Microglia are the primary participants in neuroinflammation. Sustained microglial activation promotes the release of inflammatory mediators, creating an environment that damages neurons and drives AD progression (64, 65). Under physiological conditions, both microglia and astrocytes play anti-inflammatory roles by clearing and phagocytosing Aβ and repairing damaged neurons, thereby protecting neural tissue. However, when the CNS's principal immune cells—microglia and astrocytes—become chronically activated, they release neurotoxic substances that stimulate the excessive production of inflammatory mediators namely IL-1β, IL-6, TNF-α. This heightened inflammatory response further amplifies neuroinflammation, leading to accelerated AD progression (66). In AD, excessive neuroinflammation leads to oxidative stress, and excessive oxidative damage may facilitate the formation of a cascade reaction to amplify inflammation. AD is closely associated with chronic inflammation (67). Our results demonstrated that ASO-supplementation significantly alleviated neuroinflammation, reduced systemic inflammation, and improved oxidative stress responses. This was achieved by suppressing the proliferation of microglia and astrocytes, thereby regulating central and systemic immune homeostasis in the hippocampus of AD mice.

Changes in gut microbiota structure and functionality may modulate AD development (68). Gut microbiota dysbiosis can also promote Aβ secretion, impair gastrointestinal function and the BBB, induce neuroinflammation, and lead to AD (69). Consequently, consuming foods that modulate gut microbiota structure and composition may influence the MGBA and have potential effects for AD prevention and treatment. The gut microbiota impacts host health by generating massive bioactive metabolites. SCFAs, key metabolites of gut microbiota, provide energy to intestinal epithelial cells, maintain intestinal pH, inhibit pathogenic bacteria, and contribute to immune regulation, and anti-inflammatory responses. In this study, ASO-administration increased SCFAs generation and related microbe enrichment in gut of 5 × FAD mice, and regulated gut microbiota composition and structure. FMT and sodium Buty intervention experiments also indicated that gut microbiota may provide neuroprotection by influencing gut microbiota and its metabolites. Our results demonstrated that ASO markedly enhanced the richness and diversity of gut microbiota in AD mice, resulting in the improvement of gut microbiota structural composition. This modulation indicates that ASO might provide neuroprotection via the MGBA by altering gut microbiota and their metabolites.

AD is closely linked to lipid metabolism dysfunction. It is increasingly understood that disruptions in lipid metabolism play a major role in AD pathogenesis. Brain is one of the most lipid-rich organs, with glycerophospholipids and sphingolipids serving as crucial components of neuronal membranes and myelin sheath. These lipids establish favorable conditions for protein interactions, intracellular trafficking, and functional activity (70). Sphingolipids, highly concentrated in the brain, play a crucial role in supporting the structural and functional integrity of the nervous system. Impaired sphingolipid metabolism contributes to AD, via disruption of membrane organization (71, 72). Furthermore, evidence suggests that alterations in phospholipid metabolism may be associated with AD, with reduced levels of phosphatidylethanolamine, phosphatidylinositol and phosphatidylcholine, observed in the AD brain (73, 74). Using mass spectrometry imaging techniques, researchers identified disruptions in glycerophospholipid metabolism in APP/PS1 mice aged 2 months (75). A metabolomics study detected altered glycerophospholipid metabolism in the hippocampus of male 3xTg-AD mice at 2 and 6 months (76). Significant disturbances in glycerophospholipid metabolism were observed in serum and brain from APP/PS1 and WT mice, as revealed by comparative metabolomics. Dietary lipid intake directly influences blood metabolite changes, as shown in lipid digestion and absorption.

In the present study, ASO treatment altered multiple metabolic features. Our research indicated that ASO primarily affected the biosynthesis of unsaturated FAs, glycerophospholipid metabolism, and sphingolipid metabolism. We hypothesize that the enhanced learning and memory of ASO-supplemented mice is ascribed to the combined effects of NA and essential FAs. FMT experiment was subsequently conducted to assess whether gut microbiota mediate the beneficial effects of ASO on learning and memory defcits in AD mice. Notably, FMT intervention was shown to modulated glycerophospholipid metabolism, reduced Aβ deposition, enhanced SCFAs generation, and alleviated learning and memory impairments in AD mice. The elevated presence of SCFA-producing microorganisms, especially Buty-producing bacteria in the intestinal microbiome, represents the primary mechanism by which ASO mitigates memory deficits in 5 × FAD mice. To strengthen the validation of of Buty's effects in AD mice, we performed Sodium Buty intervention experiment. The results showed that Sodium Buty intervention could modulate the composition and relative abundance of gut microbiota, and alleviate learning and memory deficits in AD mice. In conclusion, ASO administration mitigated learning and memory dysfunction in 5 × FAD transgenic mice via the microbiota-gut-brain axis. The results provide insight into the association between gut microbiota and AD. However, the present study has certain limitations. Notably, these findings are confined to an AD mouse model and remain to be confirmed in clinical settings. Future studies will aim to address these limitations through more in-depth investigations.

## Conclusions

ASO supplementation reshaped the gut microbiota composition, increased the levels of SCFAs generation and related microbe enrichment, particularly those related to Buty production, and modulated the biosynthesis of unsaturated FAs, glycerophospholipid metabolism, and sphingolipid metabolism. These changes contributed to alleviated neuroinflammation and oxidative stress, reduced Aβ deposition, and eventually improved learning and memory deficits in AD mice, exerting neuroprotective effects via the microbiota-gut-brain axis. FMT and sodium Buty intervention were shown to alleviated learning and memory dysfunction in AD mice. This study lays the groundwork for the further development of specific vegetable oils, particularly ASO, which is rich in unsaturated FAs and NA.

## Data Availability

The gut microbiomics data produced by this research are stored in the National Center for Biotechnology Information (NCBI) under the accession PRJNA1291367 and PRJNA1291372. Hippocampus transcriptomics data have been deposited at the NCBI database under the accession PRJNA1292433. Serum metabolomics data discussed herein are available in the Open Archive for Miscellaneous Data (OMIX) under the accession number: OMIX011031.
